# The C-terminal 32-mer fragment of hemoglobin alpha is an amyloidogenic peptide with antimicrobial properties

**DOI:** 10.1007/s00018-023-04795-8

**Published:** 2023-05-17

**Authors:** Lia-Raluca Olari, Richard Bauer, Marta Gil Miró, Verena Vogel, Laura Cortez Rayas, Rüdiger Groß, Andrea Gilg, Raphael Klevesath, Armando A. Rodríguez Alfonso, Kübra Kaygisiz, Ulrich Rupp, Pradeep Pant, Joel Mieres-Pérez, Lena Steppe, Ramona Schäffer, Lena Rauch-Wirth, Carina Conzelmann, Janis A. Müller, Fabian Zech, Fabian Gerbl, Jana Bleher, Nico Preising, Ludger Ständker, Sebastian Wiese, Dietmar R. Thal, Christian Haupt, Hendrik R. A. Jonker, Manfred Wagner, Elsa Sanchez-Garcia, Tanja Weil, Steffen Stenger, Marcus Fändrich, Jens von Einem, Clarissa Read, Paul Walther, Frank Kirchhoff, Barbara Spellerberg, Jan Münch

**Affiliations:** 1 Institute of Molecular Virology, Ulm University Medical Center, 89081 Ulm, Germany; 2 Institute of Medical Microbiology and Hygiene, Ulm University Medical Center, 89081 Ulm, Germany; 3 Institute of Virology, Ulm University Medical Center, 89081 Ulm, Germany; 4 Core Facility for Functional Peptidomics, Ulm Peptide Pharmaceuticals (U-PEP), Ulm University Medical Center, 89081 Ulm, Germany; 5 Core Unit of Mass Spectrometry and Proteomics, Ulm University Medical Center, 89081 Ulm, Germany; 6 Max-Planck-Institute for Polymer Research Mainz, 55128 Mainz, Germany; 7 Central Facility for Electron Microscopy, Ulm University, 89081 Ulm, Germany; 8 Computational Biochemistry, Center of Medical Biotechnology, University of Duisburg-Essen, 45141 Essen, Germany; 9 Institute of Virology, Philipps University Marburg, 35043 Marburg, Germany; 10 Institute of Protein Biochemistry, Ulm University, 89081 Ulm, Germany; 11 Laboratory of Neuropathology, Department of Imaging and Pathology, Leuven Brain Institute, KU Leuven, Leuven, Belgium; 12 Department of Pathology, UZ-Leuven, 3000 Leuven, Belgium; 13 Institute for Organic Chemistry and Chemical Biology, Center for Biomolecular Magnetic Resonance, Goethe University, 60438 Frankfurt am Main, Germany

**Keywords:** AMP, Hemoglobin fragment, Amyloid formation, Membrane disruption, Proteolytic generation

## Abstract

Antimicrobial peptides (AMPs) are major components of the innate immune defense. Accumulating evidence suggests that the antibacterial activity of many AMPs is dependent on the formation of amyloid-like fibrils. To identify novel fibril forming AMPs, we generated a spleen-derived peptide library and screened it for the presence of amyloidogenic peptides. This approach led to the identification of a C-terminal 32-mer fragment of alpha-hemoglobin, termed HBA(111–142). The non-fibrillar peptide has membranolytic activity against various bacterial species, while the HBA(111–142) fibrils aggregated bacteria to promote their phagocytotic clearance. Further, HBA(111–142) fibrils selectively inhibited measles and herpes viruses (HSV-1, HSV-2, HCMV), but not SARS-CoV-2, ZIKV and IAV. HBA(111–142) is released from its precursor by ubiquitous aspartic proteases under acidic conditions characteristic at sites of infection and inflammation. Thus, HBA(111–142) is an amyloidogenic AMP that may specifically be generated from a highly abundant precursor during bacterial or viral infection and may play an important role in innate antimicrobial immune responses.

**Supplementary Information:**

The online version contains supplementary material available at 10.1007/s00018-023-04795-8.

## Introduction

Antimicrobial peptides (AMPs) are small cationic peptides that represent a first line of defense against invading pathogens. They are highly diverse within and between species and have been reported in all living organisms, including bacteria, fungi, and humans [1, 2]. Altogether, more than 3400 AMPs have been described [3]. The predominant positive charge and the alternation between hydrophilic and hydrophobic regions in their sequences make AMPs ideally suited to bind microbial membranes [4]. Their typical mode of action is thus to integrate into the bacterial cell membrane resulting in membrane rupture and cell death. Some AMPs also enter bacteria and affect the metabolism and growth by alternative mechanisms [5, 6].

Research of the past decade showed that many AMPs can form amyloids and vice versa, that many pathological amyloids have AMP-like antimicrobial properties [7–10]. The self-assembly of peptides and proteins into amyloids has been linked to pathologies like Alzheimer’s and Parkinson’s diseases for a long time. While the oligomeric forms of the amyloids seem to be responsible for the cytotoxic or pathological effects, the monomeric and fibrillar forms are involved in the defense against microbes [7, 11, 12]. A prime example is amyloid-β, which plays a central role in Alzheimer’s disease, and was recently shown to have antibacterial effects as a monomer [13]. Moreover, amyloid-β also binds herpes virus particles and mediates antiviral effects when present in the fibrillar state [14]. Another example is β-2-microglobulin which is associated with dialysis-related amyloidosis but also possesses antimicrobial activity, especially at low pH conditions [15, 16]. Physiologically present amyloids may similarly exert antimicrobial activity, as shown for SEVI (semen-derived enhancer of viral infection), fibrils naturally present in human semen derived from the prostatic acid phosphatase (PAP) [17]. SEVI fibrils entrap bacteria and apoptotic sperm in the female reproductive tract thereby facilitating their phagocytotic uptake [18, 19]. A well-known AMP that has been shown to form fibrils is LL-37. This cathelicidin is an amphipathic, helical peptide with broad-spectrum antimicrobial activity, which self-assembles into amyloid-like protofibrils that bind DNA to activate innate immunity [7].

This functional reciprocity of amyloid fibrils and AMPs may help to identify as yet unknown amyloidogenic peptides and proteins with AMP activity. Historically, endogenous human AMPs were identified based on sequence homologies with known AMPs and the subsequent characterization of the recombinantly expressed or synthesized peptides for antibacterial, antifungal, or antiviral activity [20]. Another strategy is to directly purify AMPs from human body fluids or organs by applying chromatographic techniques in combination with functional antibacterial or antiviral assays. We and others previously used this approach and isolated novel antiviral and antibacterial peptides with often unexpected functions, by screening complex peptide/protein libraries derived from body fluids or organs [21]. Examples are EPI-X4 [22] and VIRIP [23], which were isolated from blood as inhibitors of HIV-1 infection, antitrypsin purified from lung lavage as an inhibitor of SARS-CoV-2 [24], a C-terminal fragment of human β-hemoglobin from placenta that inhibits HSV-2 infection [25], cystatin fragments that inhibit HIV and SIV infections [26], SEVI fibrils with HIV enhancing and antibacterial activity [18, 23], or the antibacterial β-2-microglobulin [16]. All of these AMPs were identified by activity-guided purification.

Since some AMPs and amyloids have strikingly similar structural and biophysical properties, we here asked whether the systematic screening of peptide libraries derived from the human body for amyloidogenic peptides would lead to the discovery of as-yet-unknown human AMPs involved in innate immunity. To achieve this, we generated a peptide library from human spleen, an important immune organ in innate and adaptive immunity [27, 28], and screened for the presence of fibrillar aggregates.

## Materials and methods

### Peptide extraction

Spleens were obtained from patients who gave their consent to the use of their organs for scientific purposes. The ethics committee at Ulm University approved the extraction and use for the production of peptide libraries (number 274/12). Spleens (9 pieces, a total of 2.3 kg wet weight) were lyophilized at − 80 °C. The dry material (457.15 g) was crushed, homogenized in 4 M acetic acid (4–12 °C) for 2 min, and then stirred (100 rpm) for 24 h at 4 °C. Subsequently, the tissue homogenate was centrifuged at 4200 rpm for 15 min at 4 °C. The pellet was discarded and the supernatant was centrifuged at 14,000 rpm using an ultracentrifuge at 4 °C, for 30 min. The supernatant was collected and filtered through a pore size of 20 μm, 8 μm, 5 μm, 3 μm, 1.2 μm and 0.45 μm respectively to remove any remaining tissue. The filtered homogenate was stored at 4 °C and then ultrafiltered (cut off 30 kDa) to remove any remaining particles or molecules larger than 30 kDa. The final sample volume was 10 liters.

### Generation of a peptide library from spleen

A volume of 10 L from the ultrafiltered sample was applied to a Source 15S cation-exchange column, equilibrated in eluent A (0.01 M HCl). To separate the substances contained in the ultrafiltrate, cation exchange chromatography was used, in which substances are separated based on their charge. For this purpose, a pH and salt gradient was determined during the HPLC, starting at pH 3 and NaCl concentration of 0.1 M, up to pH 9 and NaCl concentration of 1 M. The column was then rinsed with water/acetonitrile (95/5) and Acetonitrile 100%. The 35 fractions obtained by the cation exchange chromatography were divided into 8 pH pools (0–7). Each pH pool (0–7) was subjected to further fractionation by reversed-phase chromatography on a reversed-phase (PS/DVB) HPLC column Sepax Poly RP300 (Sepax Technologies, Newark DE, USA). In each case a sample volume of 20 ml (5% of the pH pool) was applied into the column; the elution was performed from A (0.1% TFA in water) to B (0.1% TFA in acetonitrile). Fractions were collected every 1 min (55 in total) and stored at − 80 °C.

### Peptide synthesis and recombinant expression of HBA(111–142) in *Escherichia coli*

Peptides were synthesized automatically on a 0.10 mmol scale using standard Fmoc solid phase peptide synthesis techniques with the microwave synthesizer (Liberty blue; CEM). A preloaded Resin with Serine was used and provided in the reactor. Once the synthesis was completed, the peptide was cleaved in 95% (v/v) trifluoroacetic acid (TFA), 2.5% (v/v) triisopropylsilane (TIS), and 2.5% (v/v) H_2_O. Then, the peptide residue was precipitated and washed with cold diethyl ether (DEE) by centrifugation. The peptide precipitate was then allowed to dry under vacuum to remove residual ether. The peptide was purified using reversed-phase preparative high-performance liquid chromatography (HPLC; Waters) in an acetonitrile/water gradient under acidic conditions on a Phenomenex C18 Luna column (5 mm pore size, 100 Å particle size, 250 x 21.2 mm). Following purification, the peptide was lyophilized on a freeze dryer (Labconco) for storage prior to use. The purified peptide mass was verified by liquid chromatography mass spectroscopy (LCMS; Waters).

HBA(111–142) peptide was recombinantly expressed as a fusion to maltose-binding protein including a polyhistidine tag and a cleavage site for tobacco etch virus protease using a pMal-c5X vector in *Escherichia coli* RV308. Expression was performed for 6 h at 26 °C in M9 mineral salt medium by the addition of 1 mM isopropyl β-d-1-thiogalactopyranoside. Purification was done in five steps: (a) Amylose resin high flow (New England Biolabs) chromatography, (b) Nickel–sepharose fast flow (GE Healthcare) chromatography, (c) fusion protein cleavage by overnight incubation with tobacco etch virus protease at 34 °C, (d) Nickel-Sepharose fast flow chromatography to separate HBA(111–142) from the fusion protein and maltose binding protein and (e) Source 15 RPC (GE Healthcare) reversed-phase chromatography. Afterwards, the purified protein was lyophilized using an alpha 2–4 LD plus freeze dryer (Christ). The purity of the protein was analyzed with Lithium dodecyl sulfate–polyacrylamide gel electrophoresis (Thermo Fisher Scientific). The chemical identity was demonstrated with matrix-assisted laser desorption/ionization mass spectrometry (theoretical monoisotopic mass: 3426.8 Da, experimental monoisotopic mass: 3,426.8 Da).

### Molecular mass measurement by MALDI-TOF and peptide sequencing by Edman degradation

For molecular measurement, the same sample (1 mg/ml) was dissolved in a mixture of 0.1%TFA in water/acetonitrile (1:1). Intact mass measurement was done on a REFLEX III MALDI-TOF mass spectrometer (Bruker-Daltonics, Germany). 1 µl of the sample was mixed with 1 µl of 5 mg/ml α-cyano-4-hydroxycinnamic acid matrix (dissolved in 0.1%TFA in water/acetonitrile 1:1) on a sample plate before the analysis. Measurements were performed in linear mode. Positive ions were accelerated at 20 kV, and up to 100 laser shots were automatically accumulated per sample position. The sequence of the active molecule was determined by automated Edman degradation using an Applied Biosystems 492 pulsed liquid phase sequencer equipped with an on-line 785A phenylthiohydantoin-derivative analyzer (Applied Biosystems, Darmstadt, Germany) in the Proteomics Facility, Justus Liebig University, by Dr. Guenther Lochnit.

### LC–MS/MS identification of HBA(111–142) in HB digestions

The digested samples (HB-pepsin/napsin at 20, 60, 120 min, and 24 h) were subjected to mass spectrometry analysis for the identification of HBA(111–142). A 15 μl-aliquot was used for mass spectrometry analysis as follows: the sample was measured using an Orbitrap Elite Hybrid mass spectrometry system (Thermo Fisher Scientific, Bremen, Germany) online coupled to a U3000 RSLCnano (Thermo Fisher Scientific, Idstein, Germany) employing an Acclaim PepMap analytical column (75 μm × 500 mm, 2 μm, 100 Å, Thermo Fisher Scientific, Bremen, Germany). Using a C18 μ-precolumn (0.3 mm × 5 mm, PepMap, Dionex LC Packings, Thermo Fisher Scientific, Bremen, Germany), samples were preconcentrated and washed with 0.1% TFA for 5 min. The subsequent separation was carried out using a binary solvent gradient consisting of solvent A (0.1% FA) and solvent B (86% ACN, 0.1% FA). The column was initially equilibrated in 5% B. In the first elution step, the percentage of B was raised from 5 to 15% followed by an increase from 15 to 40% B. The column was washed with 95% B and re-equilibrated with 5% B. The mass spectrometer was equipped with a nanoelectrospray ion source and distal-coated SilicaTips (FS360-20-10-D, New Objective, Woburn, MA, USA). The instrument was externally calibrated using standard compounds (LTQ Velos ESI Positive Ion Calibration Solution, Pierce, Thermo Scientific, Rockford, USA). The system was operated using the following parameters: spray voltage, 1.5 kV; capillary temperature, 250 °C; S-lens RF level, 68.9%. XCalibur 2.2 SP1.48 (Thermo Xcalibur, RRID:SCR_014593) (Thermo Fisher Scientific, Bremen, Germany) was used for data-dependent tandem mass spectrometry (MS/MS) analyses. Full scans ranging from *m*/*z* 370 to 1700 were acquired in the Orbitrap at a resolution of 30,000 (at *m*/*z* 400) with automatic gain control (AGC) enabled and set to 10^6^ ions and a maximum fill time of 500 ms. Up to 20 multiply-charged peptide ions were selected from each survey scan for collision-induced fragmentation (CID) in the linear ion trap using AGC set to 10,000 ions and a maximum fill time of 100 ms. For MS/MS fragmentation, a normalized collision energy of 35% with an activation *q* of 0.25 and an activation time of 30 ms was used. Database search (PEAKS DB) was performed using PEAKs X + studio (PRAKS Studio, RRID:SCR_022841) [29]. For peptide identification, MS/MS spectra were correlated with the UniProt human reference proteome set (Uniprot release 2020_08; 20,374 reviewed entries). Parent mass error tolerance and fragment mass error tolerance were set at 15 ppm and 0.5 Da, respectively. A maximal number of missed cleavages was set at 3. Carbamidomethylated cysteine was considered as a fixed modification and methionine oxidation as a variable modification. False discovery rates were set on the peptide level to 1%. FreeStyle™ 1.8 SP1 (Thermo Fisher Scientific, USA) was used for spectra visualization and deconvolution.

### Fibril formation and thioflavin T assay

To form fibrils, HBA(111–142) lyophilized powder was dissolved in PBS at 10 or 5 mg/ml, and agitated on a Thermoshaker for 24 h at 37 °C and 1500 rpm. Throughout this manuscript we refer to the freshly dissolved lyophilized powder as FD-HBA(111–142), and fibrillar, agitated peptide as AG-HBA(111–142). To monitor the presence of amyloid fibrils, a 2.5 mM stock solution of ThT in PBS was prepared and sterile-filtered. One µl of this solution was added to 10 µl of the peptide sample (1 mg/ml), then the sample was filled up to 100 µl using 89 µl pf PBS and incubated for 10 min in the dark, at RT. Fluorescence intensity scans were performed at an excitation wavelength of 450 nm and an emission of 450–650 nm for the spectral scanning, and 482 nm, for single wavelength analysis, using a Synergy H1 hybrid multi-mode reader (Biotek). Alternatively, to monitor the fibril formation kinetics, HBA(111–142) at different concentrations was incubated with a 25 µM ThT solution. The sample was pipetted into a well of the Corning^®^ 3575 plate and two glass beads with a diameter of 1–2 mm were added. The plate was sealed and incubated in a Synergy H1 hybrid multi-mode reader (Biotek) at 37 °C, under continuous orbital shaking (1 mm) at a frequency of 800 cmp. Fluorescence was measured at an excitation wavelength of 450 nm and an emission endpoint of 490 nm. Synergy H1 uses the Gen5 (Gen5 RRID:SCR_017317) software.

### Zeta potential

Samples were diluted in 1 ml of ddH_2_O and concentration, size distribution, or surface charge (zeta potential) was measured 3 times using a ZetaView TWIN (Particle Metrix) with the software Zetaview (Zetaview Nanoparticle Tracking Analyzer RRID:SCR_016647). Zeta potential was calculated by electrophoretic mobility of the fibril samples.

### ATR-FT–IR spectroscopy

For ATR FT–IR spectroscopy measurements, 200 µl of the respective 1 mg/ml fibril solution was lyophilized and the resulting powder was used for measurement [30]. All spectra were recorded on a Bruker Tensor 27 spectrometer with a diamond crystal as an ATR element (PIKE MiracleTM spectral resolution 2 cm^−1^). Every sample was measured with 64 scans. Data were analyzed with OriginPro (OriginLab) software (Origin, RRID:SCR_014212).

The materials and methods section for computational modeling, protease digestion experiments, SDS-PAGE, bacterial and viral assays, liposome dye leakage assays, cell culture, TEM and LSM analyses, phagocytosis assay, cell viability and hemolysis assays, as well as NMR experiments and structure calculations are available in the supplement.

## Results

### Isolation of a fibril-forming fragment of hemoglobin alpha from spleen

**Fig. 1:**
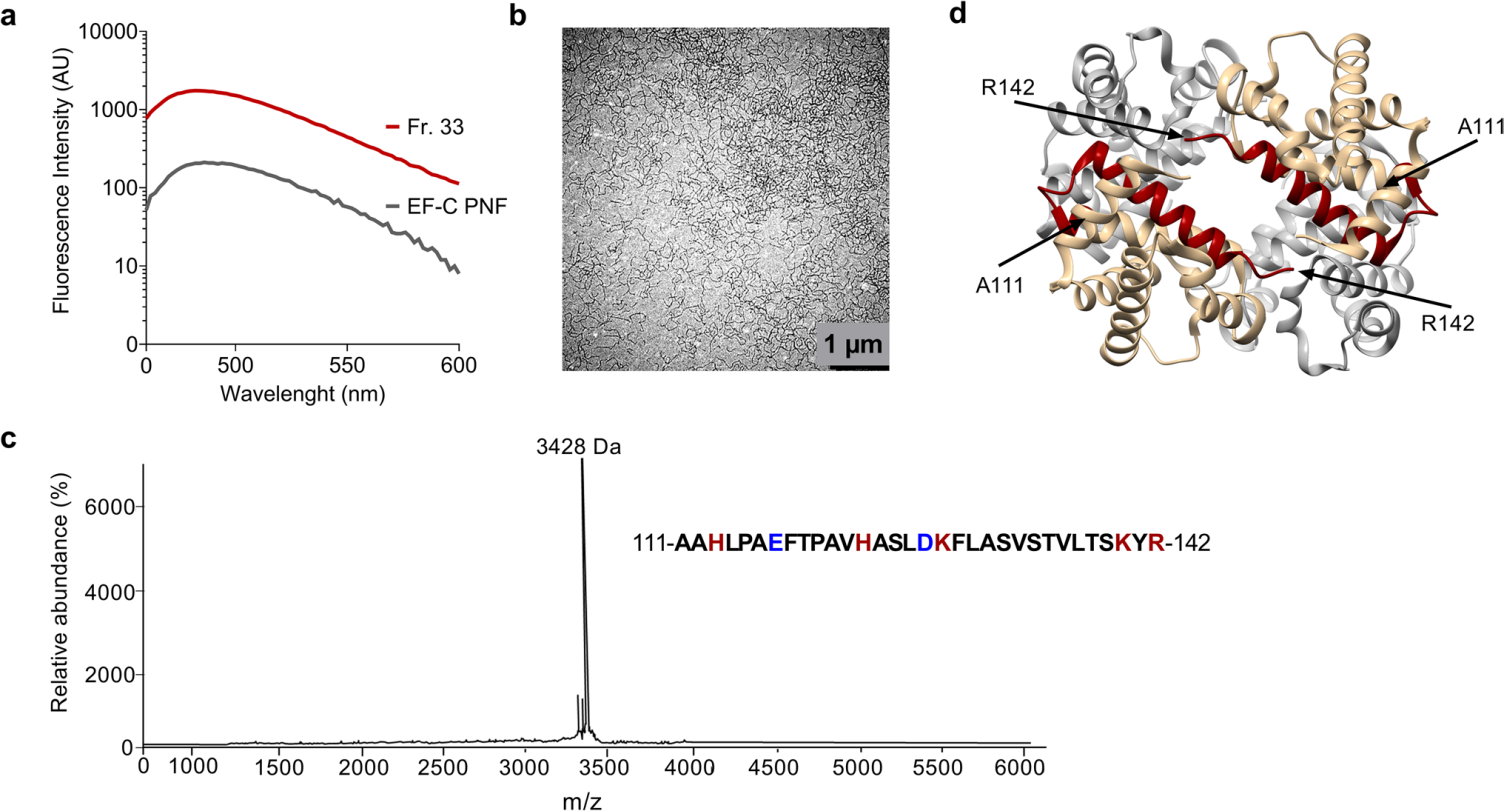
Identification of an amyloidogenic C-terminal alpha hemoglobin fragment in a human spleen-derived peptide library. **a** Fraction 33 presented a gel-like structure and was analyzed for the presence of amyloid material by ThT assay. Previously described EF-C fibrils were used as a positive control. **b** TEM images of the fraction 33. The sample was negatively stained with 2% uranyl acetate in water on copper grids and imaged with a Jeol TEM 1400. **c** MALDI-MS spectra of fraction 33. The peak corresponds to HBA(111–142) with the indicated sequence. Positively charged residues are shown in red and negatively charged in blue. **d** 3D protein structure of full-length hemoglobin consisting of two alpha and two beta subunits, depicted in brown and grey, respectively. The identified C-terminal 111–142 fragment is depicted in red (PDB 1gzx). Structures were analyzed using Chimera software

To identify endogenous amyloid-forming peptides, we generated a peptide library from 457 g of human spleen. In brief, organs derived from nine deceased donors were dried and homogenized. The homogenate was then separated by ultrafiltration, and the resulting 55 fractions contained the peptides and proteins present in the spleen with a molecular weight below 30 kDa. The lyophilized fractions were then dissolved in ddH_2_O and analyzed for the presence of peptide fibrils using the fluorescent amyloid dye thioflavin T (ThT) [32]. Fraction 33 presented a gel-like structure and exhibited a strong ThT fluorescence, higher than the positive control EF-C fibrils (Fig. 1a). EF-C (enhancing factor C) are artificial peptide nanofibrils that self-assemble in aqueous solution, and efficiently enhance retroviral gene transfer [33]. Transmission electron microscopy (TEM) analysis confirmed the presence of amyloid fibrils in fraction 33 (Fig. 1b). Mass spectrometry analysis of this fraction showed the presence of a single major peak at 3428 Da (Fig. 1c), and sequence analysis identified a 32-mer peptide that corresponds to residues 111–142 of the C-terminal region of the hemoglobin alpha subunit (HBA), termed HBA(111–142) (Fig. 1c, d).

### Biophysical characterization of HBA(111–142)

**Fig. 2:**
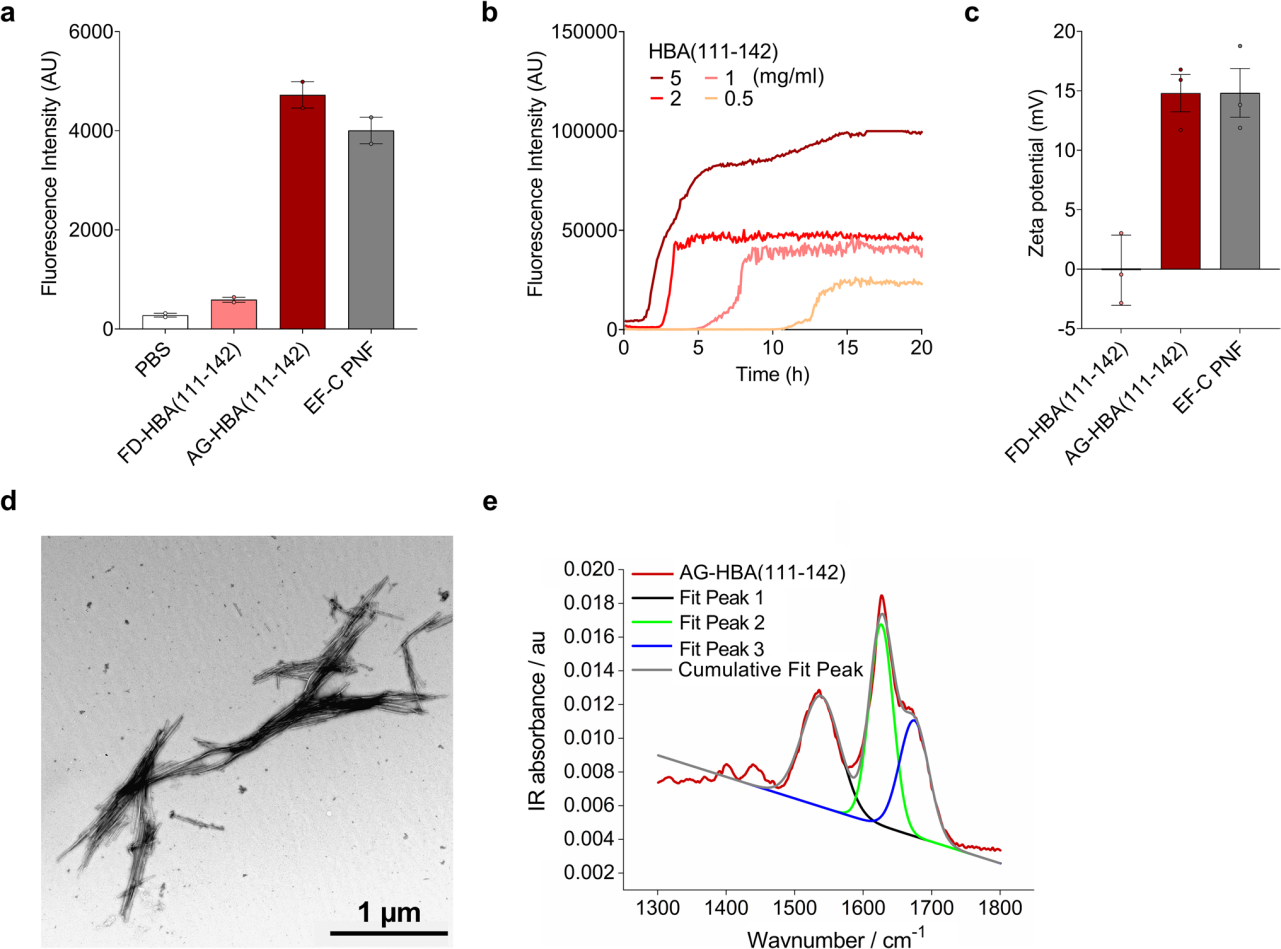
Biophysical characteristics of chemically synthesized HBA(111–142). **a** Freshly dissolved (FD) and agitated (AG) chemically synthesized HBA(111–142) and EF-C PNF as control were incubated with ThT and fluorescence intensity was measured. Shown are the means of two independent experiments performed in duplicates ± SEM. **b** Aggregation kinetics of HBA(111–142) were determined by incubating different concentrations of the peptide (0.5, 1, 2 or 5 mg/ml) with ThT. **c** Pre-formed AG-HBA(111–142) was diluted in ddH_2_O to determine the zeta potential. Positively charged EF-C fibrils were used as control. Samples were analyzed by nanoparticle tracking analysis. Shown are the means of three independent experiments each performed in duplicates ± SEM. **d** TEM images of AG-HBA(111–142). Samples were negatively stained with 2% uranyl acetate in water on copper grids and imaged with a Jeol TEM 1400. **e** ATR FT-IR spectra of AG-HBA(111–142). Shown is the peak deconvolution of the FT-IR spectra using three components with peak maxima at 1533 cm^−1^ (fit 1), 1627 cm^−1^ (fit 2), and 1674 cm^−1^ (fit 3) using gaussian shape fits. The peaks can be assigned to the amide II β-sheet, amide I β-sheet and β-turn structures, respectively. The integral ratio between fit 2 and fit 3 was used to quantify the β-sheet amount

For biophysical and functional characterization, HBA(111–142) was chemically synthesized and first analyzed for fibril formation. Agitation of the freshly dissolved peptide for 24 h at 37 °C resulted in the formation of amyloid fibrils, as shown by ThT fluorescence assays (Fig. 2a). Throughout the manuscript, we will refer as freshly dissolved (FD) HBA(111–142) to the monomeric/oligomeric peptide, and as agitated (AG) HBA(111–142) to the fibrillar peptide. The conversion rate of peptide monomers to fibrillar structures was 97.9 ± 0.4%, as determined by an amine-sensitive dye (fluorescamine), added to a filtered and non-filtered peptide sample, as described previously [34]. The lag time of fibril formation was reduced by increasing the peptide concentration, i.e. fibrils already formed after 2 h of agitation of a 5 mg/ml concentration, while fibril growth started only at 12 h in the 0.5 mg/ml solution (Fig. 2b). As expected from the net positive charge of + 1.2 and the high iso-electric point of 9.72, the Zeta potential of AG-HBA(111–142) was positive (15 mV), similar to that of EF-C PNF (Fig. 2c). TEM analysis of AG-HBA(111–142) revealed the presence of straight and short amyloid fibrils (Fig. 2d). Attenuated total reflection Fourier transform infrared spectroscopy (ATR FT-IR) and the fractional areas of the deconvoluted peaks at 1627 cm^−1^ and 1675 cm^−1^, demonstrated that the AG-HBA(111–142) peptide formed fibrils with a β-sheet secondary structure, consisting of 58% β-sheets (Fig. 2e). We next expressed HBA(111–142) in *E.coli* and found that the recombinantly produced peptide formed fibrils with similar biophysical and functional characteristics as the chemically synthesized peptide (Fig. S1). Due to the more convenient and cheaper production, all subsequent experiments were performed with the synthesized peptide.

### Generation of HBA(111–142) from full-length hemoglobin

**Fig. 3:**
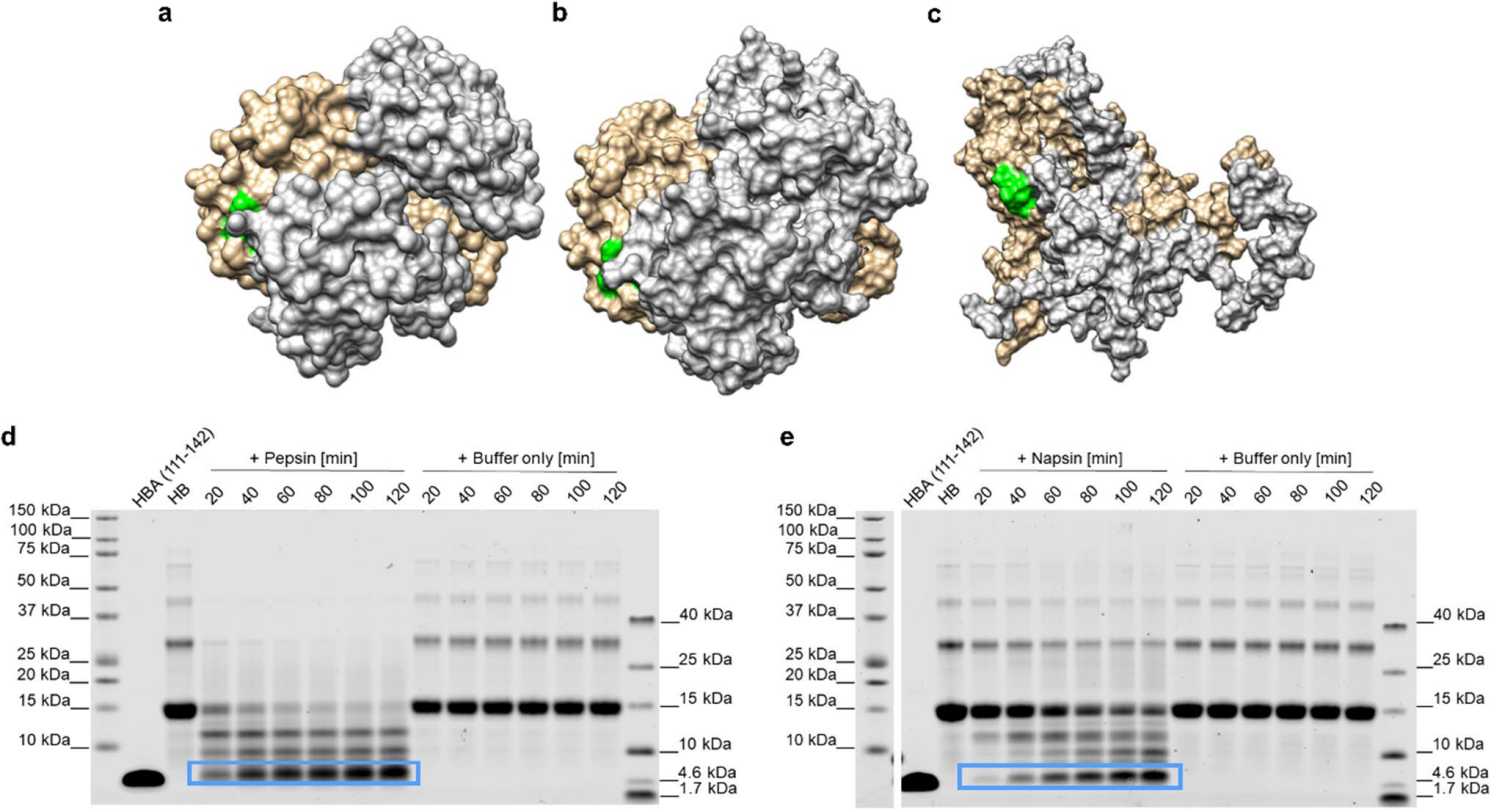
Generation of HBA(111–142) by proteolytic digestion of hemoglobin. **a** Hemoglobin crystal structure (PDB code: 2DN2 [31]). **b** Representative structure of hemoglobin from the simulations at neutral pH (7.4). **c** Representative structure of hemoglobin from the simulations at acidic pH (3.6). Two α and two β subunits are shown in brown and gray, respectively. The proposed recognition site (108–113) is displayed in green. Human hemoglobin was incubated with **d** pepsin (at pH 3.5) or **e** napsin A (at pH 3.6) at a 1:100 molar ratio, or with the respective digestion buffers without the addition of protease for 20, 40, 60, 80, 100, or 120 min at 37 °C. The reactions were separated by SDS-PAGE and total protein stained by colloidal Coomassie. The peptide of interest is marked with a blue box. Also see Fig. S3

To provide insights into how HBA(111–142) might be generated, we modeled hemoglobin at pH 7.4 (pH of blood under physiological conditions), and at pH 3.6, which occurs intracellularly in lysosomes and extracellularly at sites of infection, and is characteristic of acidic body fluids such as vaginal fluid. Since proteases usually bind and cut within a 4–6 amino acid stretch, the amino acids at positions 108–113 in HBA should be solvent-exposed to be recognized by these enzymes. To investigate a possible pH-dependent change in the accessibility of the cleavage sites by proteases, we performed all-atom, explicit-solvent Gaussian accelerated molecular dynamics (GaMD) simulations of hemoglobin at pH 7.4 (Fig. 3b) and acidic pH 3.6 (Fig. 3c). During the simulations at pH 7.4, hemoglobin preserved its structural integrity, and the recognition site (108–113) was not exposed to the solvent (Fig. 3a, b, green-colored surface). Experimental evidence suggests that, at acidic pH, the hemoglobin tetramer dissociates into the monomers [35]. Even though they do not cover the timescales of the dissociation process, our simulations at acidic pH resulted in the exposure of the 108–113 site (Fig. 3c, green-colored surface). The predicted solvent-accessible surface area (SASA; average value) of the region of interest (108–113) noticeably increased from 19.64 ± 4.16 Å^2^ at pH 7.4 to 48.87 ± 21.91 Å^2^ at pH 3.6 (Fig. S2g, j). The per-residue analysis of the SASA at the 108–113 region shows that, at pH 3.6, each of the residues in this region is more solvent exposed than at pH 7.4 (Table S1). This is also reflected in the calculated root-mean-square deviation (RMSD) of atomic positions values for hemoglobin, which, at acidic pH, displayed large fluctuations over the simulation time (RMSD of 29.42 ± 5.76 Å, Fig S2e, h). On the other hand, the simulations at pH 7.4 indicated that the overall hemoglobin structure was conserved (average RMSD: 2.29 ± 0.26 Å). Furthermore, the profile of the radius of gyration (ROG) was stable at pH 7.4 (average ROG: 40.26 ± 0.03 Å, Fig. S2f), while the hemoglobin structure was less compact at acidic pH (average ROG: 43.79 ± 0.43 Å, Fig. S2i). In addition, the predicted random coil and turn content of hemoglobin changed from 17% (pH 7.4) to 44% (pH 3.6) (Fig. S2c, d), indicating a less structured biomolecule. Taken altogether, the simulations show that at acidic pH the 108–113 region is more solvent accessible, and the hemoglobin tetramer is less compact than at neutral pH. The changes observed at pH 3.6 are expected to favor protease-mediated cleavage, thus facilitating the release of the peptide HBA(111–142).

We have previously shown that proteases with a low pH optimum proteolytically process the hemoglobin-β chain to release the antimicrobial peptide HBB(112–147) [25]. Correspondingly, we here digested full-length human hemoglobin with the acidic proteases cathepsin D, G, and E, pepsin, and napsin A at low pH, and additionally with chymase, trypsin, and lysozyme at basic pH. SDS-PAGE demonstrated that five proteases (napsin A, trypsin, pepsin, cathepsin D, and chymase) processed the monomeric globin (16 kDa) and generated fragments of lower molecular weights. However, only napsin A, pepsin, and cathepsin D generated a fragment with a size corresponding to our peptide of interest (Fig. S3a). Mass spectrometry and ESI–MS/MS analysis confirmed the generation of HBA(111–142) by pepsin and napsin A (Fig. S3d–g). HBA(111–142) was already detected after 20 min of incubation of hemoglobin with either protease, indicating that the peptide may be generated within minutes. The band intensities of HBA(111–142) increased over the 120 min while those of the hemoglobin precursor decreased (Fig. 3d, e). Importantly, HBA(111–142) remained stable even after 24 h of incubation (Fig. S3b, c). Thus, the aspartic protease pepsin, which is abundantly present in gastric juice and in low amounts in the blood [36], and napsin A, which is expressed in kidneys, lungs, and spleen [37], release HBA(111–142) under low pH conditions.

### Antibacterial activity of HBA(111–142)

**Fig. 4:**
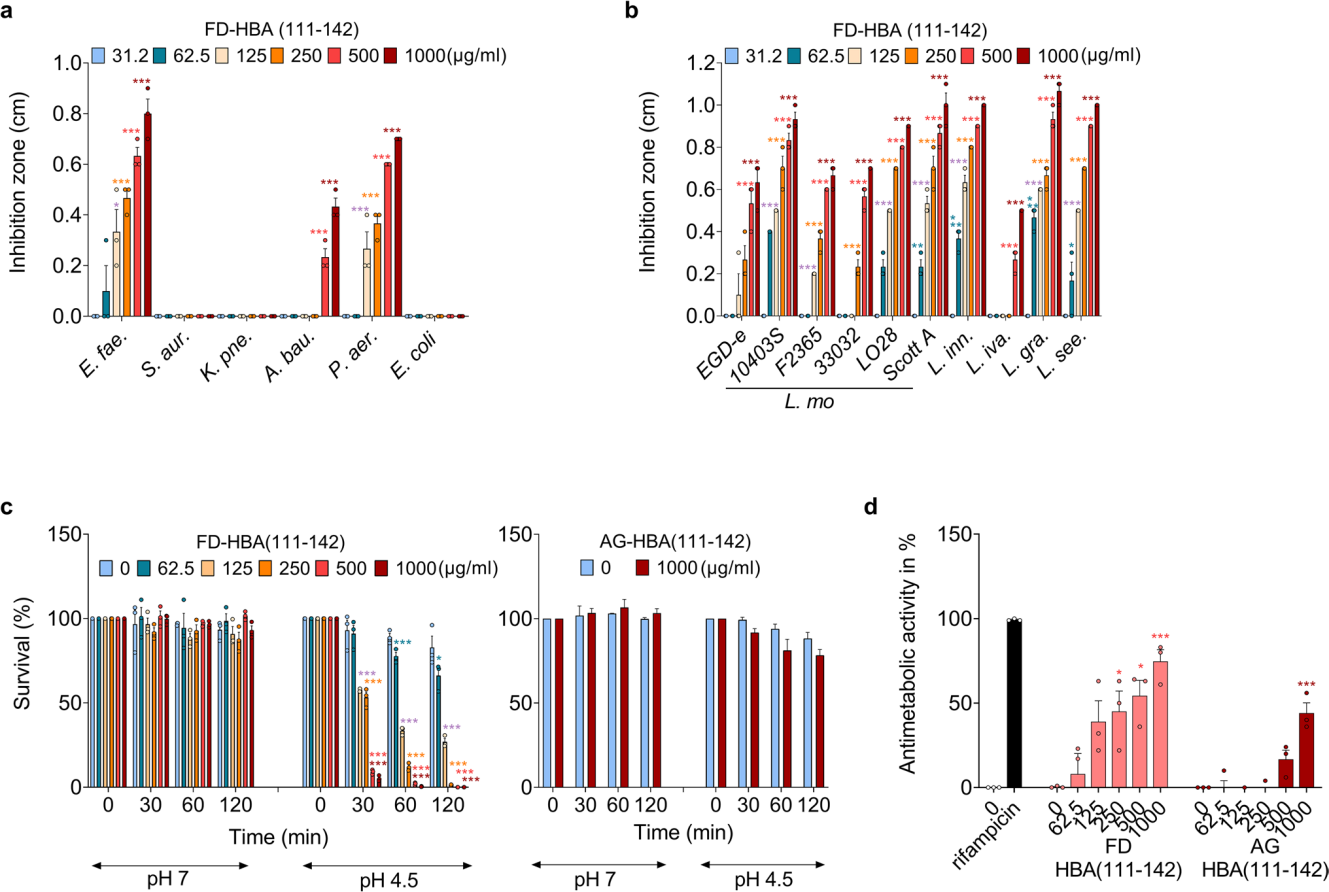
Antibacterial activity of HBA(111–142). Effect of different concentrations of FD-HBA(111–142) against the growth of **a** ESKAPE pathogens, **b** *L. monocytogenes* (*L. mo.*) strains, and other *Listeria* species in a radial diffusion assay. The peptide was dissolved in ddH_2_O. Depicted are inhibition zones in cm observed in the radial diffusion assay. **c** Effect of pH on the survival of *L. mo.* in the presence of FD- and AG-HBA(111–142). *L. mo.* was incubated with indicated concentrations of FD- and AG-HBA(111–142) at pH 7 or pH 4.5. Bacterial survival was quantified after 0, 30, 60, and 120 min by counting colony-forming units. **d** Effect of FD- and AG-HBA(111–142) against extracellular *M. tuberculosis*. The positive control, rifampicin was used at a concentration of 2 µg/ml. Data presented in a-d were derived from three independent experiments performed in singlicates and are shown as means ± SEM. Significant differences to the control were determined by one-way ANOVA followed by Bonferroni’s multiple comparison test. *p < 0.033, **p < 0.002, ***p < 0.0002

To investigate the antibacterial activity of HBA(111–142), the freshly dissolved (FD) peptide was analyzed in a radial diffusion assay for antibacterial activity against the ESKAPE panel of pathogens: *Enterococcus faecium* (*E. fae.*), *Staphylococcus aureus* (*S. aur.*), *Klebsiella pneumoniae* (*K. pne.*), *Acinetobacter baumannii* (*A. bau.*), *Pseudomonas aeruginosa* (*P. aer.*), and *Escherichia coli* (*E. col.*). Additionally, we examined *Listeria monocytogenes* (*L. mo*.) and *Mycobacterium tuberculosis*. The agitated peptide was not tested in the radial diffusion assays since fibrils do not diffuse in the agar. FD-HBA(111–142) inhibited the growth of Gram-positive *L. monocytogenes* and *E. faecium* as well as Gram-negative *P. aeruginosa* and *A. baumannii* in a dose-dependent manner (Fig. 4a). *L. monocytogenes* was inhibited at concentrations as low as 62 µg/ml. For this reason, we analyzed the activity against a larger panel of *Listeria* strains of the following species: *L. monocytogenes*, *L. innocua* (*L. inn*.), *L. ivanovii* (*L. iva.*)*, L. grayi* (*L. gra.*), and *L. seeligeri* (*L. see*,). FD-HBA(111–142) inhibited the growth of all tested *Listeria* strains (Fig. 4b).

As slightly acidic conditions are present in the radial diffusion assay, we next investigated a potential pH dependency of the antibacterial effect of FD- and AG-HBA(111–142) in bacterial growth assay. FD- and AG-HBA(111–142) were incubated with *E. coli* at pH 7 and pH 4.5 over 120 min. As shown in Fig. S4, FD-HBA(111–142) did not affect bacterial growth at pH 7 (confirming data from the radial diffusion assay, Fig. 4a) while AG-HBA(111–142) reduced growths by appr. 50%. At pH 4.5, both, freshly-dissolved and agitated HBA(111–142) entirely prevented growth of *E. coli* but did not kill the bacteria cells, as shown by the remaining living cells (CFU/ml) that were still present at 120 min (Fig. S4). Thus, at a more acidic pH, HBA(111–142) has a potent bacteriostatic activity against *E. coli*.

We next studied a potential pH dependency of the antibacterial effect of FD- and AG-HBA(111–142) in a Listeria survival assay. An overnight culture of *L. monocytogenes* was resuspended and adjusted to the pH values of 7 and 4.5. After a 30 min incubation with FD- or AG-HBA(111–142), bacterial survival was quantified by determining colony-forming units (CFU) (Fig. 4c). While no reduction in survival was observed at pH 7, after 30 min of incubation at pH 4.5 less than 50% of bacteria were viable at concentrations above 125 µg/ml of FD-HBA(111–142). AG-HBA(111–142) was tested at pH 7 and 4.5, but no reduction in *L. monocytogenes* survival was observed at either pH value (Fig. 4c). Finally, both HBA(111–142) preparations showed a dose-dependent activity against *M. tuberculosis*, killing 75% of bacteria in its FD form, and 44% when agitated (Fig. 4d). Thus, the freshly-dissolved and to a lesser extend agitated HBA(111–142) exert potent antibacterial activity, in particular at low pH.

### Mechanism of antibacterial activity

**Fig. 5:**
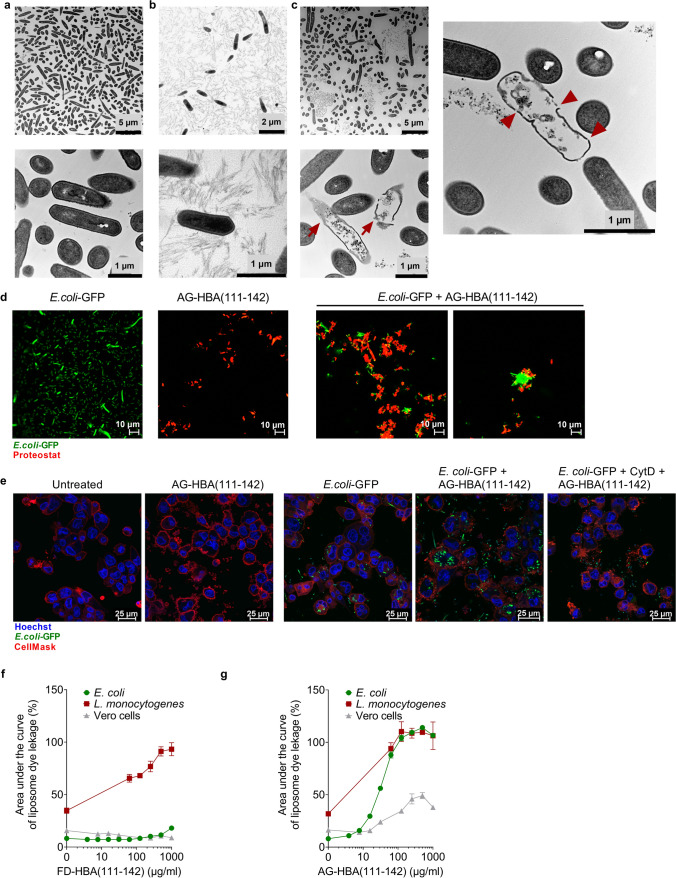
Mechanism of the antibacterial activity. *L. monocytogenes* cells were exposed to **a** buffer or 1 mg/ml of **b** AG-HBA(111–142), or **c** FD-HBA(111–142) at pH 4.5 for 30 min. Following embedding in Epon, ultra-thin sections (80 nm) were collected on copper grids and imaged with a Zeiss TEM 109 or a Jeol TEM 1400. Defect bacteria are marked with a red arrow, and holes in the cell membrane are marked with red arrowheads. **d** AG-HBA(111–142) (50 µg/ml) was stained with Proteostat^®^ amyloid staining dye (red signal) and incubated with mid-logarithmic grown *E. coli*-GFP bacteria (green signal) for 1.5 h. Results are representative of two independent experiments. Images were taken with a Zeiss LSM 710 confocal microscope. **e** Phagocytosis of *E. coli*-GFP in the presence of AG-HBA(111–142). THP-1-derived macrophages were incubated for 3 h with *E. coli*-GFP treated with 1 mg/ml AG-HBA(111–142) for 1 h at 37 °C, or PBS as a control. Cells pre-treated with 2 µg/ml phagocytosis inhibitor, Cytochalasin D (CytD) for 30 min serve as an additional control. Cell nuclei were stained with Hoechst, and the cell membrane was stained with CellMask. Samples were imaged with a Leica DMi8 confocal microscope. Liposomes filled with carboxyfluorescein were formed from bacterial or eukaryotic cells derived lipids. FD- (**f**) or AG- (**g**) HBA(111–142) were incubated with the liposomes at indicated concentrations and fluorescence increase (indicating membrane disruption) was recorded. Background signal was subtracted and normalized to fluorescent signal achieved in each well after complete liposome leakage induced by the addition of 2% (v/v) Triton X-100. Shown is the area under the curve of liposome dye leakage kinetics performed with different liposomes and compound dilutions. The area under the curve was calculated for three independent experiments performed in singlicates and shown is the mean ± SEM

The most common mode of action of AMPs is direct membrane damage by pore formation [38]. The interaction with negatively charged microbial membranes is facilitated by the cationic nature and amphipathicity of the AMP sequences. To investigate whether this is also the mechanism underlying the antibacterial activity of HBA(111–142) against *L. monocytogenes*, bacterial cultures were incubated with FD- and AG-HBA(111–142) at a pH of 4.5 for 30 min. TEM images revealed a substantial reduction of intact bacteria following the treatment with FD-HBA(111–142). While buffer- and AG-HBA(111–142)-treated bacterial cells appear intact (Fig. 5a, b), the FD- peptide disrupted the bacterial membrane, causing intracellular content leakage (Fig. 5c).

Some fibrillar AMPs have been shown to sequester bacteria and promote phagocytotic clearance [18, 39]. To clarify whether AG-HBA(111–142) may similarly bind bacteria, we performed confocal microscopy studies with GFP-labelled *E. coli.* (Fig. 4a). Fluorescence microscopy demonstrated that AG-HBA(111–142) binds and immobilizes *E. coli*, forming large bacteria aggregates with sizes of 10–20 µm (Fig. 5d), which may promote the uptake by phagocytotic cells, as previously reported [18]. To prove that AG-HBA(111–142) indeed promotes the uptake of the fibril-bacteria aggregates, THP-1 cells were differentiated into macrophages, and *E. coli*-GFP pre-incubated with 1 mg/ml AG-HBA(111–142) were added on the cells for 3 h. As a control, cells were pre-treated with the phagocytosis inhibitor Cytochalasin D (CytD) [40]. While in the absence of AG-HBA(111–142) only a few macrophages internalized *E. coli*-GFP, in the presence of AG-HBA(111–142), bacterial uptake was strongly increased, with each analyzed macrophage containing several bacterial cells (Fig. 5e). As expected, CytD prevented the uptake of any bacteria (Fig. 5e).

Finally, we analyzed if HBA(111–142) selectively targets bacterial membranes, as compared to eukaryotic membranes. For this, liposomes were formed from lipids extracted from *E. coli*, *L. monocytogenes*, or Vero eukaryotic cells. Respective liposomes with a size of 150–200 nm containing self-quenching concentrations of carboxyfluorescein were then formed by extrusion, as previously described, and incubated with FD- or AG-HBA(111–142). FD-HBA(111–142) selectively and dose-dependently disrupted *L. monocytogenes* liposomes (Fig. 5f), confirming the TEM results (Fig. 5c). No membranolytic activity was observed when the FD-peptide was incubated with *E. coli* or Vero-derived liposomes (Fig. 5f). AG-HBA(111–142) efficiently disrupted *E. coli* and *L. monocytogenes-*derived liposomes, whereas cell-derived liposomes were less affected (Fig. 5g). Of note, neither FD- nor AG-HBA(111–142) was cytotoxic in cell culture (Fig. S5a–e), or hemolytic at a concentration of up to 1 mg/ml (Fig. S5f–i), further corroborating a selectivity for disruption of prokaryotic versus eukaryotic membranes.

### Antiviral activity of HBA(111–142)

**Fig. 6:**
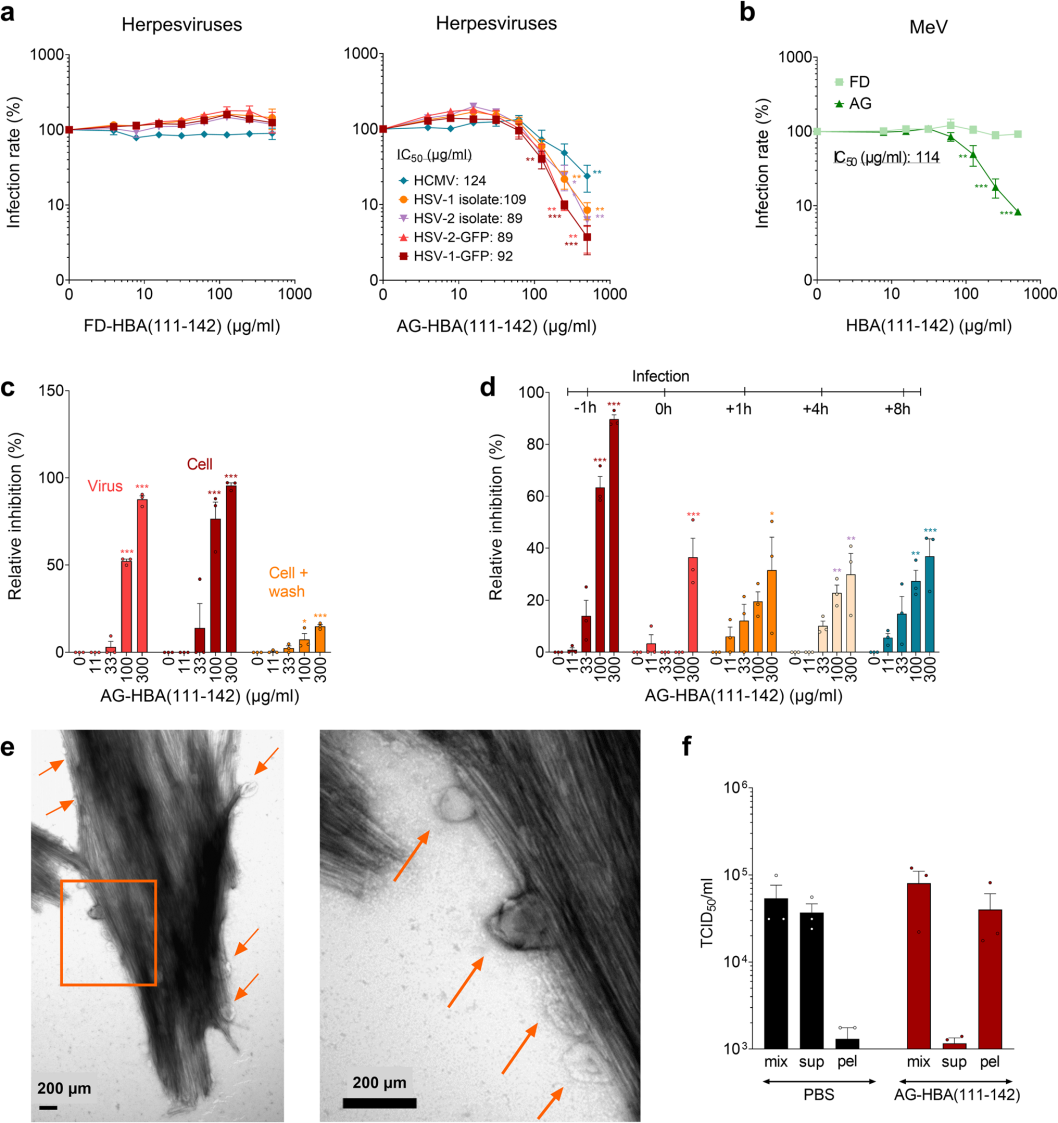
Effect of HBA(111–142) on virus infection. **a**, **b** FD- and AG-HBA(111–142) was titrated in PBS, before adding it to the cells. Following a 1 h incubation, respective target cells were infected with HSV-1, HSV-2, HCMV, and MeV. Infection rates were determined one (HSV-1, HSV-2) or two (HCMV and MeV) days post-infection. **c** HSV-2 was exposed to AG-HBA(111–142) for 1 h, then these mixtures were used to infect cells (virus treatment). Alternatively, indicated concentrations of AG-HBA(111–142) were added to cells and incubated for 1 h before the cells were either directly infected with HSV-2 (cell treatment) or first washed, supplemented with fresh medium, and then infected (wash). **d** AG-HBA(111–142) at indicated concentrations was added to the cells either 1 h prior to HSV-2 infection, at the time of infection (0 h), or 1, 4, or 8 h post-infection (with the removal of virus, washing, and addition of fresh medium). Infection rates were determined one day post-infection. **e** AG-HBA(111–142) was incubated with HSV-2 virions at a concentration of 1,000 µg/ml for 10 min. Then, the samples were fixed in PFA at a final concentration of 2%. Samples were negatively stained with 2% uranyl acetate in water on copper grids and imaged with a Jeol TEM 1400. Arrows show virions bound to the fibrils. **f** HSV-2 was incubated with PBS or 1500 µg/ml AG-HBA(111–142) for 1 h, and then an aliquot was pelleted by centrifugation. The TCID_50_/ml values of the full mixture (mix), the supernatant (sup) and the resuspended pellet (pel) were determined by infecting Vero cells. For all the cell culture experiments, concentrations indicated represent the final concentration in cell culture. Values shown are means ± SEM derived from three independent experiments performed in triplicates. Significant differences to the control were determined by one-way ANOVA followed by Bonferroni’s multiple comparison test. *p < 0.033, **p < 0.002, ***p < 0.0002

We next evaluated the antiviral activity of HBA(111–142) against different viruses. For this, target cells for the respective viruses were incubated with the freshly dissolved or agitated peptide, and subsequently infected with severe acute respiratory syndrome coronavirus type 2 (SARS-CoV-2), influenza A virus (IAV), zika virus (ZIKV), herpes simplex viruses 1 and 2 (HSV-1, HSV-2), human cytomegalovirus (HCMV) or measles virus (MeV). Infection rates were determined as described in the Supplementary Materials and Methods section. Specific virus inhibitors were included for control and resulted in the expected dose-dependent reduction of viral infection (Fig. S6). FD- and AG-HBA(111–142) had no effect on SARS-CoV-2, IAV, and ZIKV infection (Fig. S7a–c). However, AG-HBA(111–142) inhibited wildtype, as well as acyclovir-resistant HSV-1 and HSV-2 isolates in a dose-dependent manner, with IC_50_ values around 100 µg/ml (Fig. 6a). AG-HBA(111–142) was also active against HCMV and MeV (Fig. 6a, b), while the FD- peptide remained inactive against all the tested viruses (Fig. 6a, b, Fig. S7a–c).

### Agitated HBA(111–142) targets an early step of HSV infection

To determine whether the agitated peptide acts on the virions or the cells, HSV-2 was pretreated with AG-HBA(111–142) at concentrations of up to 1500 µg/ml for 1 h, before cells were infected, resulting in a further fivefold dilution of the peptide in cell culture (Fig. 6c). In addition, cells were exposed to indicated concentrations of the peptide, and then either infected directly or after a washing step that removed the fibrils. We found that the antiviral activity is determined by the concentration of the peptide in the cell culture and can also be abolished by a washing step, while the pretreatment of viral particles did not increase antiviral activity (Fig. 6c). Thus, AG-HBA(111–142) has to be present during the infection process. To clarify at which step HSV-2 infection is inhibited, FD- or AG-HBA(111–142) was either added to the cells prior to, during, or after infection. Preincubation of the cells with AG-HBA(111–142) most efficiently inhibited HSV-2 infection (Fig. 6d). This is similar to heparin, which blocks viral attachment to the cell surface (Fig. S7d, f). However, even when AG-HBA(111–142) was added 1, 4, or 8 h post-infection, HSV-2 infection rates were still suppressed by up to 40% (Fig. 6d), suggesting that the fibrils may also limit the spread of infection, presumably by binding to newly produced virions. These results were confirmed in ELVIS cells (Fig. S7h–j). Unlike the agitated form, the FD-HBA(111–142) had no anti-HSV-2 activity (Fig. S7e, g). In line with these findings, TEM confirmed that AG-HBA(111–142) binds HSV-2 virions (Fig. 6e). This finding is further supported by centrifugation experiments, where the fibril-virus mixture was mainly present in the pellet, and not in the supernatant, as it was the case for the PBS control. However, the fibrils had no virucidal activity, as the infectious titer of the virus incubated with AG-HBA(111–142) was not reduced (Fig. 6f). Thus, AG-HBA(111–142) may on the one hand sequester HSV-2 virions, thereby preventing the infection and spread of the progeny virus. On the other hand, it may also bind to the cell surface thereby preventing virion attachment and productive infection.

### Identification of the minimal active sequence in HBA(111–142)

**Fig. 7:**
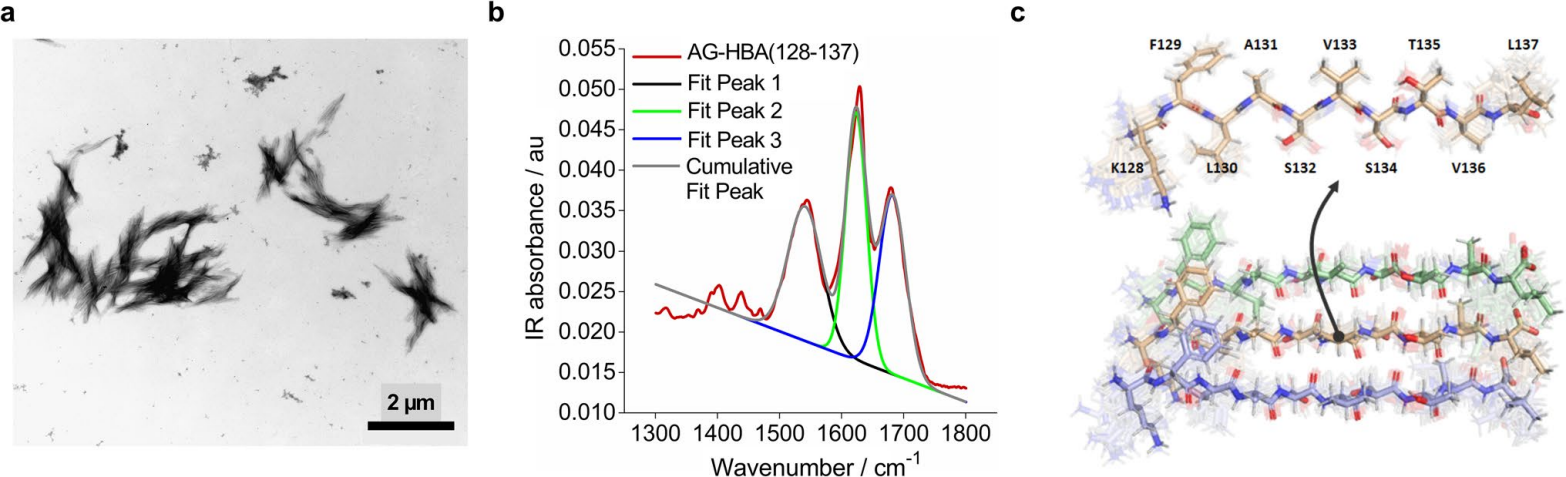
Biophysical characteristics of HBA(128–137). **a** TEM images of AG-HBA(128–137). Fibrils were negatively stained with 2% uranyl acetate in water on copper grids, and imaged with a Jeol TEM 1400. **b** ATR FT-IR spectra of AG-HBA(128–137). Shown is the peak deconvolution of the spectra using three components with peak maxima at 1,539 cm^−1^ (fit 1), 1627 cm^−1^ (fit 2), and 1674 cm^−1^ (fit 3) using gaussian shape fits. The peaks can be assigned to the amide II β-sheet, amide I β-sheet and β-turn structures, respectively. The integral ratio between fit 2 and fit 3 was used to quantify the β-sheet amount. **c** NMR-based structure of the HBA(128–137) 10-mer peptide as calculated in the context of a symmetrical in-register parallel beta-sheet (bottom). The residues are labeled on the monomeric unit (top, about 90 degrees rotated). The lowest energy structure is highlighted and the full ensemble of the best 20 structures is shown faded in the background. The Fig. was generated using PyMOL

To clarify which part of the HBA(111–142) peptide is important for fibril formation and/or anti-HSV-2 activity, we performed a structure–activity relationship (SAR) study. For peptide design, we took into consideration the isoelectric point and the prediction of aggregation propensity of the sequences as calculated by the AGGRESCAN software [41]. In each set of tests, predicted peptides were examined for their ability to form ThT-positive fibrils and to inhibit HSV-2 infection. Truncation of the C-terminal five amino acid residues, HBA(111–137), hardly affected antiviral activity, whereas truncation by 10 residues, HBA(111–131), abrogated fibril formation and antiviral activity (Table S2). N-terminal truncations of seven and 16 amino acids were tolerated, with HBA(127–137) being even more active (IC_50_ of 19.5 µg/ml) than the original peptide, HBA(111–142) peptide, with an IC_50_ of 89 µg/ml (Table S2). HBA(128–137), in which the N-terminal D residue at position 127 was deleted, showed a similar fibril forming behavior and antiviral activity. Further deletions at the N- or C-terminus resulted in a complete loss of antiviral activity (Table S2). Thus, our SAR analysis identified residues 128–137 as the minimal active sequence. The 10-mer HBA(128–137) corresponds to the predicted aggregation hotspot (127–137) with a net charge of + 1 at pH 7, and an isoelectric point (pI) of 9.91, slightly higher than the mother peptide HBA(111–142) of 9.72. The average aggregation propensity was also increased compared to HBA(111–142), from 0.08 to 0.59. Further biophysical characterization of HBA(128–137) revealed the formation of positively charged fibrils with a surface charge of + 10 mV, and a conversion rate of 86.2%. HBA(128–137) fibrils showed a classical amyloid-like phenotype (Fig. 7a), and a secondary structure of 52% β-sheets (Fig. 7b).

Finally, we performed NMR spectroscopy to get insights into the structure of HBA(128–137). Secondary structure prediction of the 10-mer peptide based on the assigned NMR chemical shifts indicated a tendential beta-strand 3D structure that matches well with the formation of fibrils (Fig. 7a). The initial structure calculations of the monomeric peptide resulted in unlikely folds with unfavorable backbone torsion angles and violations of some ROE-derived proton-proton distances; indicating that these correlations may, in fact be (partially) intermolecular. This agrees with the observed formation of a clear gel in the NMR tube (multimer formation) accompanied by perturbation of the backbone amide signal of the C-terminal leucine (L137) (Fig. S8b). The supposed ambiguous ROEs are often local (intra-residue and sequential) and would thereby indicate an in-register parallel β-sheet. Subsequent calculations in the context of such a β-sheet (restrained as a symmetrical homo trimer) allow for proper calibration of the ROE cross peaks to ambiguous distances and result in realistic structural models without violations and proper backbone torsion angles (Fig. 7c, Table S3, S4). Taken all together, we identified HBA(128–137) as the region responsible for fibril formation which allowed us to get more insights into the peptide structure and fibril forming mechanism. HBA(128–137) is the minimal active sequence against HSV-2 and may represent a potential candidate for an anti-HSV drug.

## Discussion

In this study, we screened a spleen-derived peptide library for the presence of amyloidogenic peptides and identified a C-terminal fragment of α-hemoglobin, HBA(111–142), as a novel AMP with selective antibacterial and antiviral activity. HBA(111–142) is released from the abundant hemoglobin precursor under acidic conditions, which are a hallmark of inflammation and infection [42]. Thus, HBA(111–142) may specifically be generated at sites of microbial infection and replication to act as part of the innate immune defense against invading pathogens.

Historically, AMPs have mainly been identified using sequence- or activity-guided approaches [20, 21, 24]. We here thought to discover novel endogenous AMPs by specifically searching for peptides that form amyloid structures. Amyloids are aggregates with a fibrillar morphology, a β-sheet secondary structure and the ability to be stained by particular dyes, such as ThT. Amyloids have been associated with many diseases in humans [43], but it became increasingly clear that they also have physiological functions in pigment deposition [44], hormone release and innate immunity [7, 45]. Screening a spleen-derived peptide library for ThT-reactive fractions here allowed to identify a C-terminal 32-mer fragment of hemoglobin alpha. Our biophysical and mechanistic studies confirm that HBA(111–142) forms positively charged fibrils that resemble classical amyloid fibrils rich in β-sheet structures. In addition, the HBA(111–142) peptide also has features of an AMP, like the size of about 30 residues, an overall cationic charge with positively charged lysine, arginine, and histidine residues, which get protonated in acidic environments. This may promote conformational changes and the ability to insert into bacterial membranes, possibly explaining the increased antibacterial activity of the peptide under acidic conditions [46]. Another important feature of HBA(111–142) is the presence of hydrophobic residues alternating with hydrophilic ones, which also makes the structure ideally suited for binding membranes [47].

In previous studies, the HBA(111–142) peptide has been detected in fractions derived from menstrual blood with anti-*E. coli* activity [48] and fluid of bacterially-infected wounds [49]. However, a specific antibacterial activity of HBA(111–142) has never been confirmed. In addition, HBA(111–142) is upregulated in the human cerebellum of Alzheimer’s patients [50]. At present, the in vivo concentrations of HBA(111–142) in inflamed tissues or fluids are not known. However, hemoglobin is a highly abundant and vastly present protein. While red blood cells contain a high amount of hemoglobin due to their unique structure and plasticity, it has been reported that hemoglobin is also expressed by nonerythroid cells from human tissues including lungs, neurons, retina, and endometrium [51]. Hemoglobin reaches concentrations of 150 mg/ml in the blood [52]. This means that half of this amount consists of α-hemoglobin subunits. The half-maximal concentrations of HBA(111–142) required to inhibit bacteria and viruses are in the two-digit µg/ml range, suggesting that only about 2% of the available hemoglobin needs to be digested to reach HBA(111–142) concentrations required for antimicrobial activity.

Our computational modeling analysis suggests that at low pH the protease cleavage site 108–113 in the hemoglobin alpha chain becomes more accessible than at pH 7.4, presumably allowing enzyme recognition and cleavage. Acidic pH was reported during microbe infections, when the acid–base balance in the extracellular milieu is severely challenged, leading to a pH reduction and inflammatory cytokine production [42]. Additionally, while red blood cells have a decreased lifespan in inflammation microbes can also be hemolytic, which may both promote the release of hemoglobin [53]. With decreasing pH, the hemoglobin tetramer dissociates into dimers and even monomers [35], which may result in even better exposure of the protease cleavage site, allowing efficient processing by proteases.

We showed that HBA(111–142) is generated from the hemoglobin monomer under acidic conditions by the aspartic proteases pepsin and napsin A. Pepsin is an endopeptidase secreted as pepsinogen by the gastric chief cells in the stomach wall, and activated in the form of pepsinogen by the gastric juice [54]. Pepsin has its highest activity at low pH levels, but the molecule is stable up to pH 7 [55]. The pepsinogen A gene, PGA3, has also been shown to be expressed in the brain, pancreas, or spleen [56, 57], where it may also locally generate HBA(111–142). The second enzyme shown to generate HBA(111–142) is napsin A, which is present in the lung, kidney, spleen, and placenta [37]. ELISA measurements demonstrated that napsin A is also present in human spleen tissue (data not shown). Thus, our findings suggest that, besides the generation in the normal metabolism of red blood cells, HBA(111–142) may be released either in the stomach from the digestion of alimentary hemoglobin by pepsin, or by local acidification caused by an inflammation or infection in various tissues where napsin A is present. Proteolytic processing of hemoglobin also gives rise to other fragments with antimicrobial activity, such as HBB(112–147) [25, 58]. Like HBA(111–142), the latter is also generated under acidic conditions by napsin A. Thus, proteolytic processing of the hemoglobin-α and -β subunits generates at least two distinct peptides with antimicrobial properties. It will be interesting to investigate whether both peptides may act synergistically.

Freshly dissolved HBA(111–142) inhibited the growth of several ESKAPE pathogens, such as Gram-positive *Enterococcus faecium*, Gram-negative *Pseudomonas aeruginosa*, and *Acinetobacter baumannii*. Additionally, the peptide was active against various strains of *Listeria spp.* and the highly pathogenic *Mycobacterium tuberculosis*. HBA(111–142) also showed broad activity against several other Listeria species including *Listeria ivanovii*, *Listeria seeligeri*, and *Listeria gravi*. *Listeria monocytogenes* is a human pathogenic species causing listeriosis, a serious infection characterized by febrile gastroenteritis in immunocompetent individuals, abortions in pregnant women, meningitis in newborn, and fatal infections in the elderly or immunocompromised individuals [59]. Due to the fact that it can grow at temperatures as low as 0 °C, it is considered one of the most dangerous foodborne pathogens [60]. The antibacterial activity observed was dose-dependent and the best at a low pH of 4.5. The increased activity at low pH is an advantage, as low pH values are present locally at sites of infection and inflammation [42], giving the peptide a selective activity while avoiding cytotoxicity at neutral pH values. This result is in accordance with many well-known AMPs such as LL-37, lactoferrin, or histatins that show pH-dependent antibacterial activities [61]. The mechanism underlying the antibacterial activity of the monomeric/oligomeric form of the peptide is a pH-dependent destruction of the bacterial membrane which results in cell death. This mode of action was confirmed by dye leakage studies using liposomes formed from bacteria lipids, which were selectively disrupted by the hemoglobin fragment, in contrast to liposomes resembling the eukaryotic cell membrane. In contrast to the monomeric/oligomeric peptide, the HBA(111–142) fibrils have an indirect antibacterial activity by immobilizing the bacteria, but not directly causing cell death. However, the dye leakage studies indicated that the fibrils potently disrupt bacterial liposomes. These results suggest that the fibrils can destroy the bacterial membrane, but not the bacterial cell wall, an essential protective barrier for bacterial cells. This decoration of the bacterial cell surface with fibrils results in enhanced uptake by phagocytotic cells such as macrophages or neutrophils, as previously shown for SEVI fibril and bacterial clearance in the female reproductive tract [18].

In contrast to the relatively broad antibacterial activity of FD-HBA(111–142), its antiviral activity is dependent on the formation of fibrillar structures and seems mainly restricted to herpes viruses. AG-HBA(111–142) inhibited HSV-1, HSV-2, and HCMV, which belong to the family of *herpesviridae*, and measles virus, a paramyxovirus, with IC_50_ values of around 100 µg/ml, but had no significant effect on other enveloped viruses such as IAV, ZIKV, and SARS-CoV-2. The reason(s) for this selectivity may involve a specific interaction with the viral glycoproteins or non-protein structures of the viral envelope of HSV-1, HSV-2, HCMV, and MeV. Similar to the experiments with bacteria, our data on HSV-2 shows that AG-HBA(111–142) does not act virucidal, but interacts with HSV-2 virions and the cell surface in a way that prevents viral infection and spread.

Lastly, a SAR study allowed us to identify the minimal active sequence, which encompasses residues 128–137. Similar to the original form, HBA(128–137) forms amyloid fibrils that inhibit HSV-2 infection with an IC_50_ of only 19 µg/ml. Of note, this 10-mer peptide was similarly active against acyclovir-resistant HSV-1 and HSV-2 strains and may serve as lead for further development as topically administered drug in HSV-caused labial or genital blisters.

Taken together, proteolytic processing of the highly abundant hemoglobin precursor by acidic proteases results in the generation of the antimicrobially active HBA(111–142) peptide. The peptide has been found in acidic body fluids and may play an important role in the innate immune response against invading pathogens, either by direct membranolytic activity or sequestration of pathogens.

### Supplementary Information

Below is the link to the electronic supplementary material.

Supplementary file1 (DOCX 3479 KB)

## Data Availability

The raw data supporting the conclusions of this article will be made available by the authors, without undue reservation, to any qualified researcher upon reasonable request.
