# Supplementary material for: The C-terminal 32-mer fragment of hemoglobin alpha is an amyloidogenic peptide with antimicrobial properties
Source: Cell Mol Life Sci. 2023 May 17;80(6):151. doi: 10.1007/s00018-023-04795-8 (PMC10191403; doi:10.1007/s00018-023-04795-8)
Supplement: Supplementary file 1 — Supplementary file1 (DOCX 3479 KB) [file 18_2023_4795_MOESM1_ESM.docx]

**Supplementary Material:**

**The C-terminal 32-mer fragment of hemoglobin alpha is an amyloidogenic peptide with antimicrobial properties**

Lia-Raluca Olari^1^, Richard Bauer^2^, Marta Gil Miró^1^, Verena Vogel^2^, Laura Cortez Rayas^3^, Rüdiger Groß^1^, Andrea Gilg^1^, Raphael Klevesath^2^, Armando A. Rodríguez Alfonso^4,5^, Kübra Kaygisiz^6^, Ulrich Rupp^7^, Pradeep Pant^8^, Joel Mieres-Pérez^8^, Lena Steppe^1^, Ramona Schäffer^1^, Lena Rauch-Wirth^1^, Carina Conzelmann^1^, Janis A. Müller^9^, Fabian Zech^1^, Fabian Gerbl^2^, Jana Bleher^10^, Nico Preising^4^, Ludger Ständker^4^, Sebastian Wiese^5^, Dietmar R. Thal^11,12^, Christian Haupt^10^, Hendrik R. A. Jonker^13^, Manfred Wagner^6^, Elsa Sanchez-Garcia^8^, Tanja Weil^6^, Steffen Stenger^2^, Marcus Fändrich^10^, Jens von Einem^3^, Clarissa Read^3,7^, Paul Walther^7^, Frank Kirchhoff^1^, Barbara Spellerberg^2^, and Jan Münch^1,5^*

* corresponding author: [jan.muench@uni-ulm.de](mailto:jan.muench@uni-ulm.de)

^1^ Institute of Molecular Virology, Ulm University Medical Center, 89081 Ulm, Germany

^2^Institute of Medical Microbiology and Hygiene, Ulm University Medical Center, 89081 Ulm, Germany

^3^ Institute of Virology, Ulm University Medical Center, 89081 Ulm, Germany

^4^ Core Facility for Functional Peptidomics, Ulm Peptide Pharmaceuticals (U-PEP), Ulm University Medical Center, 89081 Ulm, Germany

^5^ Core Unit of Mass Spectrometry and Proteomics, Ulm University Medical Center, 89081 Ulm, Germany

^6^ Max-Planck-Institute for Polymer Research Mainz, 55128 Mainz, Germany

^7^ Central Facility for Electron Microscopy, Ulm University, 89081 Ulm, Germany

^8^ Computational Biochemistry, Center of Medical Biotechnology, University of Duisburg-Essen, 45141 Essen, Germany

^9^ Institute of Virology, Philipps University Marburg, 35043 Marburg, Germany

^10^ Institute of Protein Biochemistry, Ulm University, 89081 Ulm, Germany

^11^ Laboratory of Neuropathology, Department of Imaging and Pathology, Leuven Brain Institute, KU Leuven

^12^Department of Pathology, UZ-Leuven, 3000 Leuven, Belgium

^13^ Institute for Organic Chemistry and Chemical Biology, Center for Biomolecular Magnetic Resonance, Goethe University, 60438 Frankfurt am Main, Germany

**Supplementary Materials and Methods:** pages 2-8

**Supplementary Figures S1-S8:** pages 9-16

**Supplementary Tables S1-S4:** pages 17-19

**Additional References** pages 20-21

**Supplementary Materials and Methods:**

**Computational details**

The coordinates of hemoglobin were taken from a reported high-resolution crystal structure (PDB ID: 2DN2, resolution 1.25 Å)[1]. The protonation states of titrable residues at pH 7.4 and pH 3.6 were assigned using the H++ web-server[2] and visual inspection. The resultant structures were minimized (10,000 conjugate gradient + 10000 steepest descent cycles) to remove any close contacts or clashes. The AMBER software suite (Assisted Model Building with Energy Refinement) [3] was used with the updated heme parameters from the literature[4]. The protein was simulated with the AMBER force field (modified ff99SB)[5]. Counterions were added to maintain the overall electroneutrality of the systems, which were simulated in a cubic water box of explicit solvent (TIP3P)[6] using the particle mesh Ewald method (PME) for long-range electrostatics[7]. The simulation systems were then heated at room temperature for 40 ps (NVT) followed by an equilibration step of 10 ns (NPT). Finally, Gaussian accelerated molecular dynamics (GaMD) simulations[8] were performed (NPT ensemble) in explicit solvent conditions, with three replicas of 300 ns each (total of 900 ns for each setup), for the production runs of both systems. A cut-off of 8 Å was used for the non-bonded interactions. The Cpptraj tool[9] was used to monitor the RMSD and ROG fluctuations throughout the simulation time. Representative structures from the simulations at pH 3.6 and pH 7.4 were generated with the Cpptraj tool using the hierarchical agglomerative clustering method with a cutoff distance of 3 Å between any identified clusters[10]. The centroids of the dominating clusters were chosen as the representative structures shown in the manuscript. The population of the dominating cluster at neutral pH was 95.39%. Due to the dynamic behavior displayed by hemoglobin at acidic pH, leading to large RMSD fluctuations, the population of the dominating cluster at pH 3.6 was 41.37%. The Naccess facility[11] was employed to compute the solvent-accessible surface area (SASA). The last 100 ns of each replica were used for reporting the average RMSD, ROG, and SASA values.

**Protease digestion experiments**

Proteases used were chymase (Sigma C8118), cathepsin D (Sigma SRP6415), cathepsin G (RP-77525), cathepsin E (Biovision 7842), pepsin (Roche 10108057001), trypsin porcine pancreas (Sigma T0303-1G), lysozyme from chicken egg white (Sigma L6876), napsin A (RND 8489-NA). Digestion experiments were carried out with purified human hemoglobin (Sigma H7379) and recombinant or purified proteases. 100 mg hemoglobin (ca. 1.56 nmol) were digested with chymase (in Tris-HCl 0.05 M, pH 8.0/0.26 M NaCl), cathepsin D, G and E (in 0.2 M citrate buffer, pH 5.0), pepsin (in 20 mM sodium acetate buffer, pH 3.5), trypsin (in 0.1 M Tris–HCl, with 10 mM CaCl_2_, pH 8), lysozyme (in 10 mM Tris-HCl, pH 8) or napsin A (in 0.2 M NaCl, 0.1 M sodium acetate, pH 3.6). All proteases were used at a 1:100 molar ratio (15 pmol), and reactions were incubated at 37 °C for 2 h.

**SDS-PAGE**

For SDS-PAGE, samples were incubated with Protein Loading Buffer (LiCor) and reducing agent and heated at 70 °C for 10 min. 10 µg of hemoglobin were loaded per lane. After running, the gel was washed for 5 min with ultrapure water, then fixed with 50% MeOH/7% acetic acid for 15 min and washed three times 5 min with ultrapure water. Staining was then performed with GelCode Blue (colloidal coomassie) overnight. Destaining was done with ultrapure water until the background appeared clear, the gel then imaged in a LiCor Odyssey system running on Image Studio (Li-Cor Image Studio Software, RRID:SCR_015795).

**Radial diffusion assay**

Bacteria were incubated in liquid THY broth (Todd-Hewitt Broth [Oxoid, CM0189B] supplemented with 0.5% yeast extract [BD, Miami, USA, 212750]) at 37 °C overnight in a 5 % CO_2_ atmosphere. Cells were pelleted by centrifugation and washed once in 10 mM PBS. The resulting pellet was suspended in 10 mM PBS and the optical density was determined spectrophotometrically at 600 nm (OD_600nm_). 2 × 10^7^ bacteria were mixed with 1 % agarose dissolved in 10 mM PBS and the mixture was poured into a petri dish. After cooling for 30 min at 4 °C, 2-3 mm holes were punched into the agarose plate and 10 µl of HBA(111-142) of the desired concentration was filled into the holes followed by incubation for 3 h at 37 °C in ambient air. The agarose layer was overlaid with 10 ml of a 1 % agarose solution containing 3% tryptic soy broth (TSB) dissolved in 10 mM PBS and the inhibition zones in cm were determined after the plates were incubated for 16-18 h at 37 °C in a 5 % CO_2_ atmosphere. Bacteria strains used: *Enterococcus faecium* vancomycin-resistant strain Van A, *Staphylococcus aureus* multi-dug resistant (MRSA) strain F-182, *Klebsiella pneumoniae* strain K6, *Acinetobacter baumannii* strain 2208, *Pseudomonas aeruginosa* strain Boston 41501, *Escherichia coli* strain ESBL, *Listeria innocua* CIP 107775, *Listeria ivanovii* CIP 7842T*, Listeria grayi* CIP 68.18T, *Listeria seeligeri* SLCC 3954, and *Listeria monocytogenes* strains: EGDe, 10403S, F2365, 33032, LO28, and Scott A.

**Survival assay**

*Listeria monocytogenes* (ATCC Cat# BAA-679/EGD-e) was grown in THY broth, while *E. coli* (BSU 1286) was cultivated aerobically in lysogeny broth (LB-Miller) till mid-logarithmic phase at 37 °C in a 5% CO_2_ atmosphere. An equivalent of OD_600nm_ of 0.1 was centrifuged and the pellet was suspended in PBS adjusted to pH 7 or pH 4.5. A volume of 90 µl of the bacterial suspension was mixed with 10 µl FD- or AG-HBA(111-142) of the desired concentration and incubated at 37 °C. After 0, 30, 60, and 120 min incubation, samples were spread on blood agar plates to determine the colony forming units (CFU) at each time point. The survival rate was calculated in comparison to the CFU present at the beginning of the experiment (t = 0 min).

**Activity against extracellular mycobacteria: ^3^H-Uracil proliferation assay**

The activity of freshly dissolved and agitated HBA(111-142) against virulent *Mycobacterium tuberculosis* (*Mtb*) strain H33rV was determined by measurement of RNA synthesis after incorporation of radioactively-labeled 5.6-^3^H-Uracil as described[12]. Briefly, 2 × 10^6^ sonicated*Mtb* were incubated with FD- or AG-HBA(111-142)in middlebrook 7H9 broth. 2 µg/ml rifampicin served as control. After 72 h, ^3^H-Uracil (0.3 μCi/well) was added, followed by incubation for an additional 18 h. Afterwards, *Mtb* were inactivated by treatment with 4% paraformaldehyde (PFA) for 30 min and transferred onto glass fiber filters (Printed Filtermat A, PerkinElmer) using a 96-well based Filtermat Harvester (Inotech). Fiber filters were dried and sealed with a sheet of solid scintillant wax (MeltiLex, PerkinElmer). Radioactivity was measured using a β-Counter (Sense Beta, Hidex). Antimicrobial activity (%) was calculated as counts per minute (cpm) of the treated sample/cpm of the untreated sample × 100.

**Folch extraction**

Lipids were extracted from live bacteria or Vero E6 eukaryotic cells using the Folch method[13]. Briefly, bacterial cells grown in overnight cultures (16-18 h) or Vero E6 cells from a confluent T-175 cm^3^ cell-culture flask were harvested by centrifugation and resuspended in 1 ml of 2:1 (v/v) chloroform/methanol mixture and vortexed 5 × 1 min. Then, 200 µl ddH_2_O was added and the samples were centrifuged for 7 min at 1,000 × g to induce phase separation. The lower phase containing the lipids was carefully extracted and moved in a glass vial. The liquid was removed by drying under a nitrogen gas steam. The lipid amount was quantified by measuring the glass vial before and after the addition of lipids.

**Liposome dye leakage**

Liposome leakage assay was performed as previously described[14]. Liposomes for dye-leakage assay were prepared by thin-film hydration & extrusion. Lipids previously extracted or commercially purchased (*E. coli* polar extract, Avanti lipids) were hydrated by adding 1 ml 50 mM 5(6)-carboxyfluorescein prepared in 50% PBS (resulting in a solution iso-osmolar to PBS) and adjusted to pH 7.4 with NaOH, yielding an approximate total lipid concentration of 3-5 mM. The glass vials were shaken at 70 °C, 180 rpm, for 3 h. Small unilamellar vesicles were then prepared by 25 × extrusion through 0.2 μm polycarbonate membranes (Nuclepore Track-Etched Membrane, Whatman, Maidstone, USA) in a Mini Extruder (Avanti Polar Lipids) on a heating platform at 70 °C. Free dye was removed by 2 × size-exclusion filtration using PD midiTrap Sephadex G-25 columns (GE Healthcare, Buckinghamshire, UK) and liposomes were then quantified by nanoparticle tracking analysis (NTA) using a ZetaView (Particle Metrix, Inning, Germany). Liposome preparations were diluted in PBS and 1-2 × 10^9^/well added to plates in 80 μl volume. Fluorescence intensity was measured at an excitation of 493 nm and emission at 517 nm, with a Synergy H1 plate reader (Biotek, Winooski, USA) using the Gen5 (Gen5 RRID:SCR_017317) software. The baseline was established by measuring fluorescence for 5 min, 20 μl of compounds then added and plates incubated for 1 h at 37 °C with measurements every 1 min. Maximum intensity (100% dye release) was then measured by adding Triton X-100 to 2% final concentration and again measuring for 5 min.

**Cell culture**

Vero E6 (Cercopithecus aethiops derived epithelial kidney) cells were purchased from ATCC® (ATCC Cat# CRL-1587, RRID:CVCL_0574) and grown in Dulbecco’s modified Eagle’s medium (DMEM, Gibco) which was supplemented with 2.5% heat-inactivated fetal calf serum (FCS), 100 units/ml penicillin, 100 µg/ml streptomycin, 2 mM L-glutamine, 1 mM sodium pyruvate, and 1x non-essential amino acids. Caco-2 (human epithelial colorectal adenocarcinoma) purchased from ATCC® (ATCC Cat# HTB-37, RRID:CVCL_0025) were grown in the same media but with a supplementation of 10% FCS. ELVIS cells (Enzyme-Linked Virus-Inducible System – ELVIS™), also from ATCC® (ATCC Cat# CRL-12072, RRID:CVCL_1914) are genetically engineered baby hamster kidney cells that encode a lacZ gene, which is expressed upon infection via the viral transactivator ICP10[15]. Human foreskin fibroblasts (HFF) were kindly provided by Jens von Einem (Institute of Virology, Ulm University Medical Center). Human lung epithelial carcinoma (A549) and human acute monocytic leukemia cells, THP-1 were purchased from ATCC® (ATCC Cat# CRM-CCL-185 and Cat# 30-2001, RRID: CVCL_0006 and CVCL_0023). MDCK (NBL-2) cells were purchased from ATCC® (ATCC Cat# CCL-34, RRID:CVCL_0422) and grown in DMEM which was supplemented with 0.2 % bovine serum albumin (BSA), 2 mM L-glutamine, 100 U/ml penicillin, 100 µg/ml streptomycin, 25 mM HEPES buffer and 1 µg/ml tosylsulfonyl phenylalanyl chloromethyl ketone (TPCK)-trypsin. ELVIS, HFF, and A549 cells were grown in DMEM supplemented with 2 mM L-glutamine, 100 units/ml penicillin, 100 μg/ml streptomycin, and 10% heat-inactivated FCS. THP-1 cells were grown in Roswell Park Memorial Institute (RPMI) supplemented with 2 mM L-glutamine, 100 units/ml penicillin, 100 μg/ml streptomycin, and 10% heat-inactivated FCS. All cells were grown at 37 °C/5 % CO_2_ humidified incubator.

**Effect of HBA(111-142) on HSV-1 and HSV-2 infection**

HSV-1 and -2 clinical isolates were kindly provided by Prof. Michel (Institute of Virology, Ulm University Medical Center). Acyclovir-resistant strains carry frameshift mutations in the thymidine kinase. Recombinant eGFP-encoding Herpes-Simplex-Virus 2 (Strain 333) was kindly provided by Patricia Spear (Northwestern University, USA), and HSV-1-GFP (Strain F) was provided by Benedikt Kaufer (Free University of Berlin; Germany). Virus stocks were generated by infecting 70-80% confluent Vero E6 cells in 175 cm^3^ cell-culture flasks in 30 ml cell medium. 48 h later virus was collected by centrifuging the cell supernatant to remove cell debris for 3 min at 300 × g. Virus stocks were stored at -80 °C.

For the infection assay in ELVIS cells, 5,000 ELVIS cells were seeded the day before. Before infection, the cell medium was removed and 80 µl of X-vivo cell medium supplemented with 2 mM L-glutamine, 100 units/ml penicillin, and 100 µg/ml streptomycin was added. 10 µl of FD- or AG-HBA(111-142) were added to the cells and incubated for 1 h at 37 °C prior to infection with 10 µl HSV-2. One day post-infection, the rates of infection were measured by Gal‐Screen β‐Galactosidase Reporter Gene Assay System for Mammalian Cells (Thermo Fisher Scientific) and the Orion II microplate luminometer (Berthold, Bad Wildbad, Germany) operating with the software Simplicity (Simplicity, RRID:SCR_022809).

For the infection assay in HFF cells, 10,000 HFF cells were seeded the day before. Before infection, the cell medium was removed and 80 µl of X-vivo cell medium supplemented with 2 mM L-glutamine, 100 units/ml penicillin, and 100 µg/ml streptomycin was added. Cell treatment experiments were performed by adding 10 µl of FD- or AG- HBA(111-142) to the cells and incubating for 1 h at 37 °C prior to infection with 10 µl HSV-2. Similarly, virus treatment experiments were done by mixing 35 μl of the peptide sample with 35 μl of HSV-2 for 1 h at 37 °C. Then, 20 μl of the peptide-virus mix were added to each well. One day post-infection, cells were fixed in 4% PFA, and GFP+ cells were counted using FACS analysis and the software CytExpert (CytExpert, RRID:SCR_017217).

In the time of addition experiments in HFF and ELVIS cells, HSV-2 was incubated with the cells for 2 h to allow infection and cells were washed three times with PBS, to remove the virus left in the supernatant. Then, fresh medium was added on the cells. HBA(111-142) or controls were added to the cells either 1 h before the HSV-2 infection (similar to cell treatment), in the same time with the virus (0 h), or at the indicated timepoint after washing the virus. For the experiment performed in HFF cells, infection rates were determined one day post-infection, by counting GFP+ cells using FACS analysis and the software CytExpert (CytExpert, RRID:SCR_017217), and two days post-infection by Gal‐Screen β‐Galactosidase Reporter Gene Assay System for Mammalian Cells (Thermo Fisher Scientific) and the Orion II microplate luminometer (Berthold, Bad Wildbad, Germany) operating with the software Simplicity (Simplicity, RRID:SCR_022809), for the ELVIS cells. In the ELVIS cell experiment, HBA(111-142) and controls were added 48 h post-infection, meaning 1 h before the readout, as an additional control.

For the experiments in ELVIS cells, HSV-1-GFP, HSV-2-GFP, and clinical isolates of HSV-1 and HSV-2 resistant to acyclovir were used at a multiplicity of infection (MOI) of 0.025. For the experiments in HFF cells, HSV-2-GFP was used at a MOI of 0.1.

For the Median Tissue Culture Infectious Dose (TCID_50_) experiment, 6,000 Vero E6 cells were seeded the day before, and 80 µl agitated HBA(111-142) at indicated concentrations, or the PBS control were incubated with 80 µl HSV-2-GFP virus at an MOI of 0.1 for 1 h at 37 °C. Then, the fibril-virus mixture was centrifuged for 10 min, 2,000 rpm at 4 °C. The supernatant (sup) was removed from the pellet, and the pellet was dissolved in the 160 µl medium (pel). Then, a 10-fold dilution series was prepared and used to infect the cells. 7 dpi infected wells were identified by light microscopy, the cell viability was measured via an MTT assay and the TCID_50_/ml was calculated according to Reed-Muench[16].

**Effect of HBA(111-142) on HCMV Infection**

HCMV wild type virus strain TB40E was kindly provided by Jens von Einem (Ulm University Medical Center). For the infection assay, 10,000 HFF cells per well were seeded. The following day the cell medium was removed and replaced with 80 μl of MEM cell medium supplemented with 100 units/ml penicillin, 100 μg/ml streptomycin, 2 mM glutamine, and 1% non-essential amino acids (NEA). Cell treatment was performed by adding 10 μl FD- or AG-HBA(111-142) to the cells and incubating them for 30 min at 37 °C prior to infection with 10 μl undiluted HCMV TB40E. The infected cells were incubated for 4 h at 37 °C. The cells were then washed three times with cell medium before adding 100 μl MEM medium supplemented with 100 units/ml penicillin, 100 μg/ml streptomycin, 2 mM glutamine, 1% NEA, and 10% FBS. One day post-infection, the infected cells were fixed with 4% PFA and stained against anti-IE1/2 hybridoma antibody 63-27[17] and 4′,6-diamidino-2-phenylindole (DAPI). Cell imaging was performed by using the Zeiss Axio Observer Z1 fluorescence microscope and the AxioVision Imaging System (RRID:SCR_002677). The infection rates were determined by calculating the ratio of IE1/2 positive cells to the total number of cells (DAPI-positive).

**Effect of HBA(111-142) on MeV Infection**

MeV-GFP (vac2 strain) was constructed and kindly provided by Christian Pfaller and Konstantin Sparrer[18]. MeV was propagated by harvesting confluent Vero E6 cells of a T-175 cm^3^ cell-culture flask and pelleted at 1,600 rpm for 3 min at 4 °C. Afterwards, the cell pellet was resuspended in MeV-GFP reaching a MOI of 0.01. Infected cells were incubated for 1 h at 37 °C with gently shaking every 10-15 min. Afterwards, they were diluted in cell medium and the cell suspension was divided into T-175 cm^3^ cell-culture flasks. Incubation took place at 37 °C until cytopathic effect (CPE) visible as syncytia formation was detected. Then, the media was replaced by Opti-MEM in which cells were harvested by detaching. The cell suspension was sonificated for 10 min to disrupt the cells leading to the release of the virions into the supernatant. After, the cell debris were pelleted (1,600 rpm, 3 min, 4 °C) and supernatant was stored at -80 °C as aliquots of 200 µl each. To determine the anti-MeV effect of HBA(111-142), A549 cells were seeded at a density of 20,000 cells. On the day of infection, media was removed and cells were washed once with PBS before the addition of 80 µl X-Vivo cell medium supplemented with 2 mM L-glutamine, 100 units/ml penicillin, and 100 μg/ml streptomycin per well. FD- and AG-HBA(111-142) were serially diluted in PBS and added to the cells. After an incubation period of 1 h at 37 °C, cells were infected with MeV-GFP at a MOI of 0.1. Two days post-infection, cells were fixed in 4% PFA, and GFP+ cells were counted using FACS analysis and the software CytExpert (CytExpert, RRID:SCR_017217).

**Effect of HBA(111–142) on SARS-CoV-2 Infection**

Viral isolate BetaCoV/France/IDF0372/2020 (#014V-03890) was obtained through the European Virus Archive global. Virus was propagated by inoculation of 70% confluent Vero E6 in 75 cm² cell culture flasks with 100 µl SARS-CoV-2 isolates in 3.5 ml serum-free medium containing 1 µg/ml trypsin. Cells were incubated for 2 h at 37 °C, before adding 20 ml medium containing 15 mM HEPES. Cells were incubated at 37 °C and supernatant harvested on day 3 post-inoculation when a strong CPE was visible. Supernatants were centrifuged for 5 min at 1,000 × g to remove cellular debris, and then aliquoted and stored at -80 °C as virus stocks. The infectious virus titer was determined as plaque-forming units (PFU/ml). To determine the effect of HBA(111-142) on SARS-CoV-2 infection, 30,000 Caco-2 cells were seeded. The next day, 44 µl DMEM and 18 µl 5-fold titrated compound was added. After 2 h incubation at 37 °C, cells were infected with 18 µl SARS-CoV-2 (MOI 0.009). Twodays later, infection was quantified by an enzyme-based immunodetection assay against the SARS-CoV-2 spike protein as previously described[19].

**Effect of HBA(111-142) on IAV Infection**

Influenza strain A/PR/8/34 (H1N1; PR8) was purchased from ATCC® and propagated in MDCK cells. Therefore, MDCK cells were inoculated with PR8 (MOI 0.005), which was diluted in cDMEM (DMEM supplemented with 0.2 % bovine serum albumin (BSA), 2 mM L-glutamine, 100 units/ml penicillin, 100 µg/ml streptomycin, 25 mM HEPES buffer and 1 µg/ml tosylsulfonyl phenylalanyl chloromethyl ketone (TPCK)-trypsin). After 1 h incubation, the inoculum was removed by two times washing with PBS and afterwards cDMEM was added. After two days, the cell suspension was harvested, sonicated for 10 min, and frozen at -80 °C. The next day cell suspension was thawed on ice and afterwards centrifuged at 4 °C and 300 × g for 15 min. Supernatants supplemented with 0.5 % BSA were frozen at -80 °C.

To determine the titer of IAV stocks, MDCK cells were seeded in 12-well plates one day prior to infection. Cell culture supernatants were removed, cells were washed with PBS and 100 μl of serially diluted IAV stocks from 1:10^4^ to 1:10^9^ were added with 250 μl cDMEM. Infected cells were incubated for 1 h at 37 °C with regular shaking steps in between before 2 ml of overlay medium (cDMEM, 0.01% DEAE Dextran, 0.1% NaHCO_3_, 0.6% Avicel RC 581) were added to the cells. Three days post-infection, supernatants were removed and cells were fixed with 4% PFA for 1 h at RT. Cells were washed with PBS and incubated in Crystal violet staining solution (0.5% Crystal violet, 30% Ethanol) for 10 min at RT. Cells were washed with ddH_2_O and dried until plaques were counted. The virus titer was calculated as plaque-forming units per ml (PFU/ml).

For testing anti-IAV activity, 20,000 Caco-2 cells were seeded one day prior infection. Cells were incubated with FD- and AG-HBA(111-142), and neuraminidase inhibitor oseltamivir phosphate for 30 min at 37 °C before IAV was added resulting in MOI 0.0007. Two days post-infection, infection rates were determined by MUNANA (2′-(4-methylumbelliferyl)-α-D-N-acetylneuraminic acid)) assay, quantifying neuraminidase activity in cellular lysates. For this, cells were washed with PBS, lysed in 1% Triton X-100 and 1:2 diluted in MES buffer (32.5 mM MES monohydrate, 4 mM CaCl_2_ dihydrate). 20 µl sample was incubated with 30 µl of MUNANA substrate (100 µM) and incubated for 4 h at 37 °C and 190 rpm. 150 µl stop solution (0.1 M glycine in 25% ethanol) was added to the reaction before neuraminidase activity was determined using a Synergy H1 (Biotek) imaging reader (360 nm excitation and 450 nm emission) with the software Gen5 (Gen5 RRID:SCR_017317).

**Effect of HBA(111–142) on ZIKV Infection**

Virus stocks of ZIKV MR766, a ZIKV strain isolated from a sentinel rhesus macaque in 1947[20] were generated as described above for HSV. For the infection assay, 6,000 Vero E6 cells were seeded the day before. Cells were treated with 10 µl of compounds for 10 min at 37 °C prior to infection with ZIKV MR766 at a MOI of 0.15. Two days later, infection rates were determined with a cell-based ZIKV immunodetection assay. Cells were washed with PBS and fixed with 4% PFA for 20 min at RT. Cell permeabilization was performed with cold methanol for 5 min at 4 °C, and washed with PBS. Afterward, cells were incubated with mouse anti-flavivirus antibody 4G2 (Absolute Antibody Cat# Ab00230-2.0, RRID:AB_2715504) in antibody buffer (0.3% Tween®20, 10% FCS in PBS) for 1 h at 37 °C, washed 3 times with washing buffer (0.3% Tween®20 in PBS), and incubated with horseradish peroxidase(HRP)-coupled anti-mouse antibody (Thermo Fisher Scientific Cat# A16066, RRID:AB_2534739) (1:20,000) for 1 h at 37 °C. After 4 washing steps with washing buffer, 3,3',5,5'-Tetramethylbenzidine (TMB) substrate (Medac) was added. Following an incubation of 5 min at RT, the reaction was stopped with 0.5 M sulfuric acid, and absorption was measured at 450 nm and baseline corrected at 650 nm using an ELISA microplate reader (Molecular Devices) with the software SoftMax (SoftMax Pro Data Acquisition and Analysis Software, RRID:SCR_014240).

**Negative staining and TEM imaging**

Negative staining was performed by applying 5 µl of the samples to glow discharged 300 mesh copper grids with a carbon-coated formvar film. After 5 min adsorption at RT, three washing steps with a series of three drops of distilled water and one staining step with 2% uranyl acetate in water were performed. The excess of the 2% uranyl acetate solution was removed using filter paper. The samples were then air-dried for 1-2 h. Samples were visualized using a Jeol-1400 transmission electron microscope operated at 120 kV.

**TEM imaging of bacteria**

*L. monocytogenes* (ATCC BAA-679/EGD-e) cells, grown until the mid-exponential growth phase, were harvested by centrifugation and resuspended in 5% TSB dissolved in 0.9% NaCl (pH 4.5). 5 × 10^9^ cells[21] were treated with 1 mg/ml FD-HBA(111-142) for 1 h at 37 °C followed by centrifugation. The pelleted cells were fixed with 2.5% glutaraldehyde containing 1% saccharose in 0.1 M PBS (pH 7.3). Samples were washed three times with PBS and post-fixed in 2% aqueous osmium tetraoxide. After dehydrating the samples in a graded series of 2-propanol, they were block-stained with 1% uranyl acetate in ethanol and embedded in Epon. Ultra-thin sections (70 nm) were collected on copper grids, contrasted with 0.3% lead citrate in water for one minute, and imaged with a Zeiss TEM 109 or with a Jeol TEM 1400.

**TEM imaging of HSV-2 and fibrils**

HSV-2-GFP was produced as described above. The fresh virus from 4 × 175 cm^3^ cell-culture flasks was harvested and pooled. Then, the virus was divided into 4 × 37 ml tubes and concentrated by ultracentrifugation at 39,742 × g for 2 h at 4 °C. The supernatant was removed and 50 µl MNT buffer (30 mM MES, 100 mM NaCl, 20 mM Tris in ddH_2_O) was added to the pellets and incubated overnight at 4 °C. The next day, the resuspended pellets were pooled and 180 µl of the concentrated virus was layered over an Optiprep™ density gradient (Sigma Aldrich) containing 4 fractions (15-25-40-54% Optiprep™), each in a volume of 2.5 ml. After an ultracentrifugation step at 26,000 × g for 2 h at 4 °C, the fractions were collected in separate tubes. Then, collected fractions were concentrated with a last ultracentrifugation step at 80,000 × g for 2 h at 4 °C. The supernatant was removed and 100 µl MNT buffer was added to the pellets and incubated overnight at 4 °C. To check in which fraction the infectious virus particles were present, the resuspended pellet from each fraction was added to Vero E6 cells seeded the day before 12,000 cells/well. Two days post-infection the cells were visually examined using light microscopy, and virus infection was observed only in the 15% Optiprep™ fraction. AG-HBA(111-142) with the 30 µl of the concentrated purified virus at a final concentration of 1 mg/ml. This mixture was incubated for 10 min at 37 °C and then fixed with PFA for 30 min at RT and 30 min at 37 °C at a final PFA concentration of 2%. Negative staining of samples and TEM imaging was performed as described above with the adaptations of applying 7 µl of the sample to the copper grid, 5 min of incubation, and one staining step with 2% uranyl acetate for 5 s.

**Laser Scanning Microscopy of *E. coli*-GFP and AG-HBA(111-142)**

To stain the fibril samples for imaging, the ProteoStat® Amyloid Plaque Detection Kit was used. Briefly, a proteostat dye staining solution was prepared by mixing 100 µl of 10× buffer, 900 µl of water, and 1 µl of detection reagent. Of a 2.5 mg/ml fibril stock solution, 4.8 µl was added to 55.2 µl of the prepared staining solution and incubated in the dark for 15 min at RT before imaging. *E. coli*-GFP plasmid pAKgfp1 was purchased from Addgene[22] (Addgene plasmid # 14076; http://n2t.net/addgene:14076; RRID:Addgene_14076). To visualize the interaction between *E. coli*-GFP and AG-HBA(111-142) bacteria were cultured at 37 °C/5% CO_2_ overnight in Lysogeny Broth medium following centrifugation and resuspension in PBS. The bacterial cell density was determined at 600 nm to estimate bacterial cell count/ml. An estimation of 8 × 10^8^ bacteria/ml was assumed for OD_600_=1[23]. Bacteria were adjusted to the desired concentrations and incubated with fibrils for 1.5 h at 37 °C in a thermomixer with 300 rpm agitation. After fixation with 4% PFA, 15 µl of each sample were transferred to 18-well Ibidi slides. Samples were imaged using a Zeiss LSM 710 confocal microscope and the software Zen (Black Zen, RRID:SCR_018163).

**Phagocytosis of *E. coli*-GFP in the presence of AG-HBA(111-142)**

To study the uptake of *E. coli* in the presence of AG-HBA(111-142), 150,000 THP-1 suspension cells were seeded in RPMI medium containing 10% heat-inactivated FCS, 100 units/ml penicillin, 100 µg/ml streptomycin, 2 mM L-glutamine, and 100 mM phorbol-12-myristate-13-acetate (PMA). Next day, the medium was replaced to PMA-free medium. After 24 h, the medium was replaced with antibiotic-free RMPI medium. As a control, cells were pre-treated with 2 µg/ml cytochalasin D for 30 min. *E. coli*-GFP at MOI of 10 were incubated with 1 mg/ml AG-HBA(111-142), or PBS as a control, for 1 h at 37 °C, under agitation at 300 rpm. Then, the mixtures were added on cells and incubated for 3 h. Afterwards, cells were fixed in 4% PFA for 30 min at RT. Cell nuclei were stained with Hoechst (1:2,000 dilution in PBS) for 30 min at RT, and then CellMask (1:1,000 in PBS) for 5 min at RT. Samples were imaged with a Leica DMi8 confocal microscope (Leica).

**Colorimetric 3-(4, 5-dimethylthiazol-2-yl)-2,5-diphenyltetrazolium bromide (MTT)- based cell viability assay**

Cells were seeded one day prior in 96-well flat-bottom plates. The next day, the medium was removed and 90 µl of X-vivo medium (supplemented with 2 mM L-glutamine and 100 units/ml penicillin, 100 μg/ml streptomycin) was added. FD- and AG-HBA(111-142) was serially diluted and 10 μl of each dilution was added to the cells. Cell viability was quantified two days post-treatment with the MTT-based assay. The medium was removed and 90 μl PBS and 10 μl MTT (5 mg/ml) solution were added per well. Following a 2.5 h incubation time at 37 °C, the supernatant was discarded and formazan crystals were dissolved in 100 μl 1:1 DMSO-EtOH solution. Absorption was measured at 450 nm and the baseline was corrected at 650 nm using a Vmax microplate reader (Molecular Devices) with the software SoftMax (SoftMax Pro Data Acquisition and Analysis Software, RRID:SCR_014240).

**Hemolysis assay**

10 ml blood, obtained from vein puncture was centrifuged for 10 min, 1,000 × g and 4 °C. The pelleted erythrocytes were washed three times and diluted 1:10 in PBS. HBA(111-142) either freshly dissolved or agitated was diluted in PBS and incubated with 10 µl of the erythrocyte suspension. After 1 h of incubation at 37 °C and shaking at 500 rpm, the samples were centrifuged for 5 min at 1,000 rpm and 4 °C. 100 µl of the supernatants were transferred to transparent 96-well plates. The absorption of released hemoglobin in the supernatants was measured at 405 nm using a Vmax microplate reader (Molecular Devices) with the software SoftMax (SoftMax Pro Data Acquisition and Analysis Software, RRID:SCR_014240).

**Measurement of napsin A in spleen tissue by dot blot**

Freeze-dried human spleen tissue was obtained from UPEP and grinded using mortar and pestle. The resulting powder was alternatively dissolved in PBS or in Tris buffer (20 mM Tris-HCl, 150 mM NaCl, 1 mM CaCl_2_, 1 mM EDTA at pH 7) and sonicated for 1 min (50% amplitude, pulsing 15 s on and off). Afterwards, vacuum-assisted filtration was performed using a 0.45 µm membrane. 25 µl of 1:10 dilutions of these samples were transferred as spots onto a methanol-activated Immobilon-FL PVDF membrane (Merck). Napsin was detected with polyclonal anti-huNapsin A Rabbit IgG (R and D Systems, Cat# AF8489, RRID:AB_2924264). As a loading control, β-Actin was detected with β-Actin mouse monoclonal antibody (Cell Signaling Technology Cat# 3700, RRID:AB_2242334). Both antibodies were 1:1,000 diluted in blocking buffer after overnight blocking with LiCor Odyssey Blocking Buffer (TBS) involving gentle agitation. Primary antibodies were stained with LiCor secondary antibodies diluted 1:10,000 in blocking buffer for 1 h at RT. This fluorescence-based detection was performed with IRDye 800 CW goat anti-rabbit (LI-COR Biosciences Cat# 926-32211, RRID:AB_621843) and IRDye 680RD goat anti-mouse (LI-COR Biosciences Cat# 926-68070, RRID:AB_10956588) using a Li-Cor Odyssey CLx system running on Image Studio (Li-Cor Image Studio Software ver 5.2, RRID:SCR_015795).

**NMR experiments and structure calculations**

The NMR experiments of the HBA(128-137) 10-mer peptide (dissolved in 90/10% H_2_O/D_2_O) were conducted at a temperature of 298 K on a Bruker Avance III 850 MHz spectrometer, equipped with a 5 mm QXI (^1^H,^13^C,^15^N,^31^P,^2^H) probe with z-gradients. The NMR spectra were acquired and processed using Topspin versions 3.5 - 4.1 (TopSpin RRID:SCR_014227) (Bruker Biospin). For the chemical shift assignment and structure determination, the following experiments were conducted: homonuclear 2D ^1^H^1^H-ROESY (80, 100, 120, 140, 160, and 180 ms mixing time), 2D ^1^H^1^H-TOCSY (80 ms mixing time, mlev17), 2D ^1^H^1^H-COSY (with double quantum filter) and heteronuclear 2D ^1^H^13^C-HSQC (multiplicity edited), 2D ^1^H^13^C-HMBC (optimized on long-range couplings) and 2D ^1^H^15^N-HSQC at natural abundance. The NMR structure calculations were performed using ARIA 1.2 with CNS 1.1 (Crystallography and NMR System, RRID:SCR_014223)[24, 25]. Customized protocols were used to perform the structure calculation in the context of a homotrimer using symmetry and hydrogen bond restraints for an in-register parallel beta-sheet and thereby allowing proper calibration of the rotating-frame Overhauser effect (ROE) crosspeak intensities to the corresponding proton-proton distances (ambiguous contribution of equivalent intra- and intermolecular atoms which ranges up to about 50% of the cross-peak volume) including the correction of spin-diffusion. The four 2D ^1^H^1^H-ROESY spectra (100, 120, 140, and 160 ms mixing time), used for the structure calculation, were manually peak picked and assigned using Sparky 3.114 (Sparky, RRID:SCR_014228)[26]. Ample backbone dihedral angle restraints (for the beta-strand) have been included that were a priori predicted from the NMR chemical shifts by TALOS-N (TALOS-N, RRID:SCR_022800)[27] and are well in agreement with preceding calculations where only ROEs were used. For each iteration (0-7) in which 100 structures were calculated, the ROE distance restraints were recalibrated by ARIA based on the 20 lowest energy structures. The violation tolerance was progressively reduced to 0.1 Å in the last iteration (8) in which 400 structures were calculated. For the structure calculations, the standard four-stage simulated annealing protocol was used with cartesian angle dynamics. The high temperature stage consisted of 10,000 steps at 10,000 K. This was followed by refinement and cooling down stages: 8,000 steps at 2,000 K, 5,000 steps to 1,000 K, and 10,000 steps to 50 K. During the SA protocol the force constant for the distance restraints was set to 0, 10, 10 and 50 kcalmol^-1^Å^-2^ for the successive stages. The final 20 lowest energy structures were further refined in explicit water[28]. The Fig. for the NMR-based structural model of HBA(128-137) was generated using PyMOL (PyMOL, RRID: SCR_000305).

**Supplementary Figures**

**
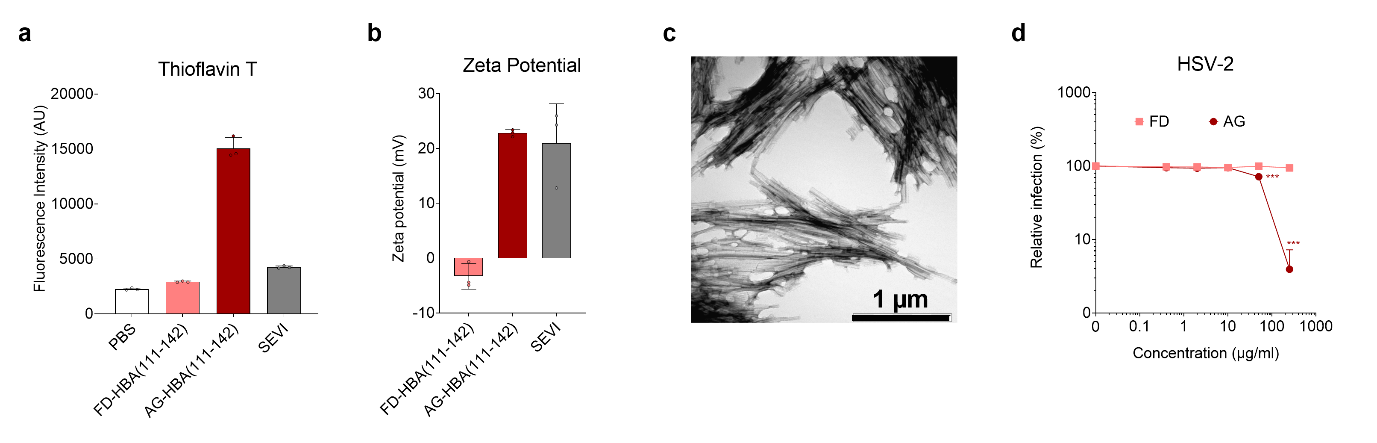
Fig. S1** **Characterization of rrecombinantly expressed HBA(111-142).** **(a)** Freshly dissolved (FD) and agitated (AG) recombinantly expressed HBA(111-142) and SEVI fibrils as control were incubated with ThT and fluorescence intensity was measured. Shown are the means of one experiment performed in triplicates ± SD. **(b)** Pre-formed AG-HBA(111-142) was diluted in ddH_2_O to determine the zeta potential. Positively charged SEVI fibrils were used as control. Samples were analyzed by nanoparticle tracking analysis. Shown are the means of one experiment performed in triplicates ± SD. **(c)** TEM images of AG-HBA(111-142). Fibrils were negatively stained with 2% uranyl acetate in water on copper grids and imaged with a Jeol TEM 1400. **(d)** FD- and AG-HBA(111-142) were titrated in PBS, before the addition to the cells, resulting in the indicated final concentrations on cells. Following a 1 h incubation, cells were infected with HSV-2-GFP. Infection rates were determined two days post-infection by measuring the mean fluorescence intensity of GFP expression using a Synergy H1 reader. Values were baseline-subtracted and normalized to cells infected without the addition of compound. Shown is the mean of one experiment performed in triplicates ± SD. Significant differences in **(d)** were determined by one-way ANOVA followed by Bonferroni’s multiple comparison test. *p< 0.033, **p< 0.002, ***p<0.0002.


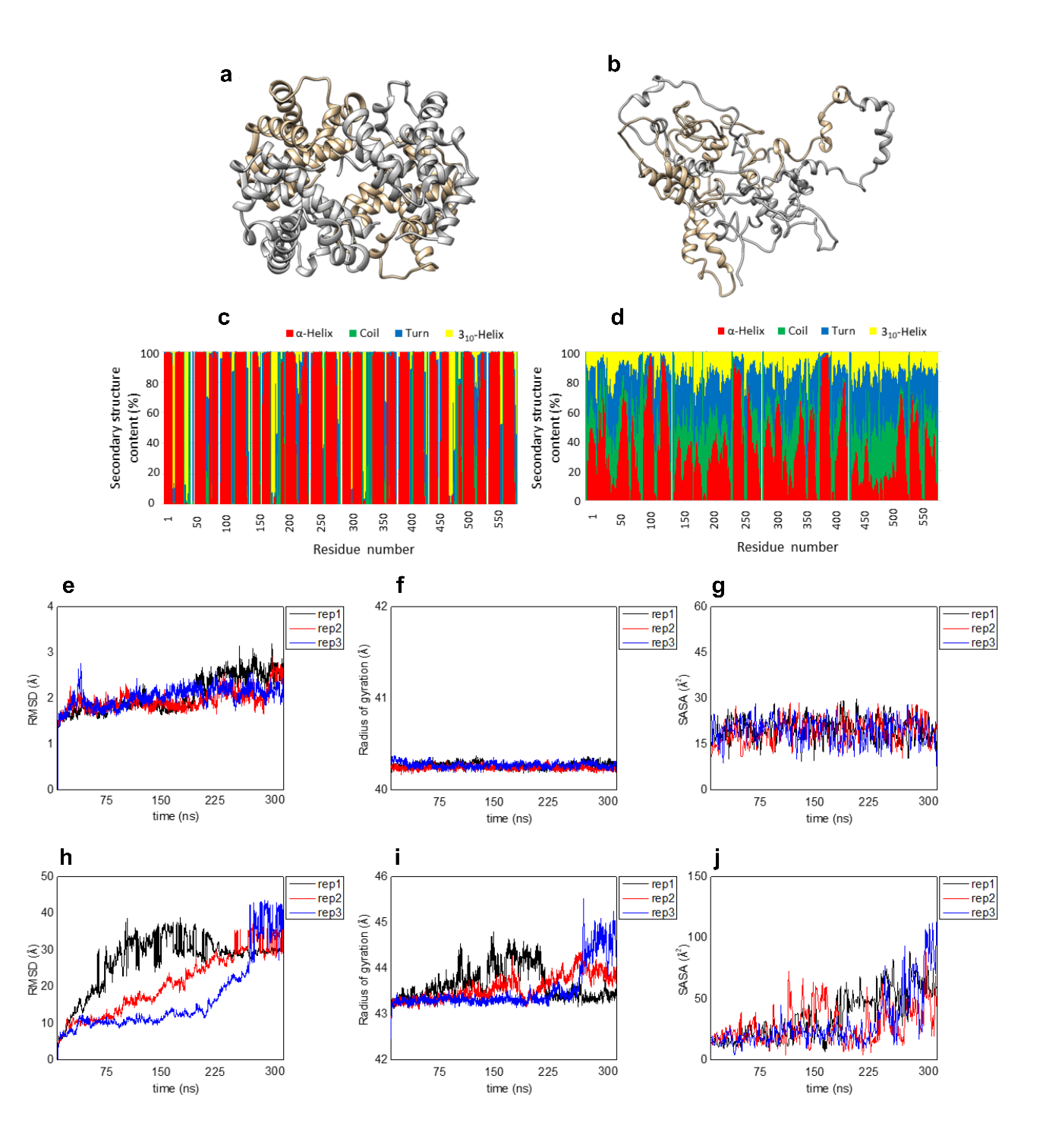
**Fig. S2 Structural characteristics of hemoglobin at pH 7.4 and 3.6 and selected parameters from the Gaussian accelerated molecular dynamics simulations. (a)** Representative structure of hemoglobin at pH 7.4. **(b)** Representative structure of hemoglobin at pH 3.6. Secondary structure content per amino acid at **(c)** pH 7.4 and (**d)** at pH 3.6. e-g correspond to pH 7.4: **(e)** RMSD fluctuations **(f)** Profile of radius of gyration (ROG) **(g)** Change in the SASA value (for residues 108 to 113) as a function of simulation time (ns). h-j correspond to pH 3.6: **(h)** RMSD fluctuations at acidic pH **(i)** ROG profile at acidic pH **(j)** Change in the SASA value (for residues 108 to 113) as a function of simulation time (ns). In all cases, blue, red and black lines represent independent replicas of the simulations.

**
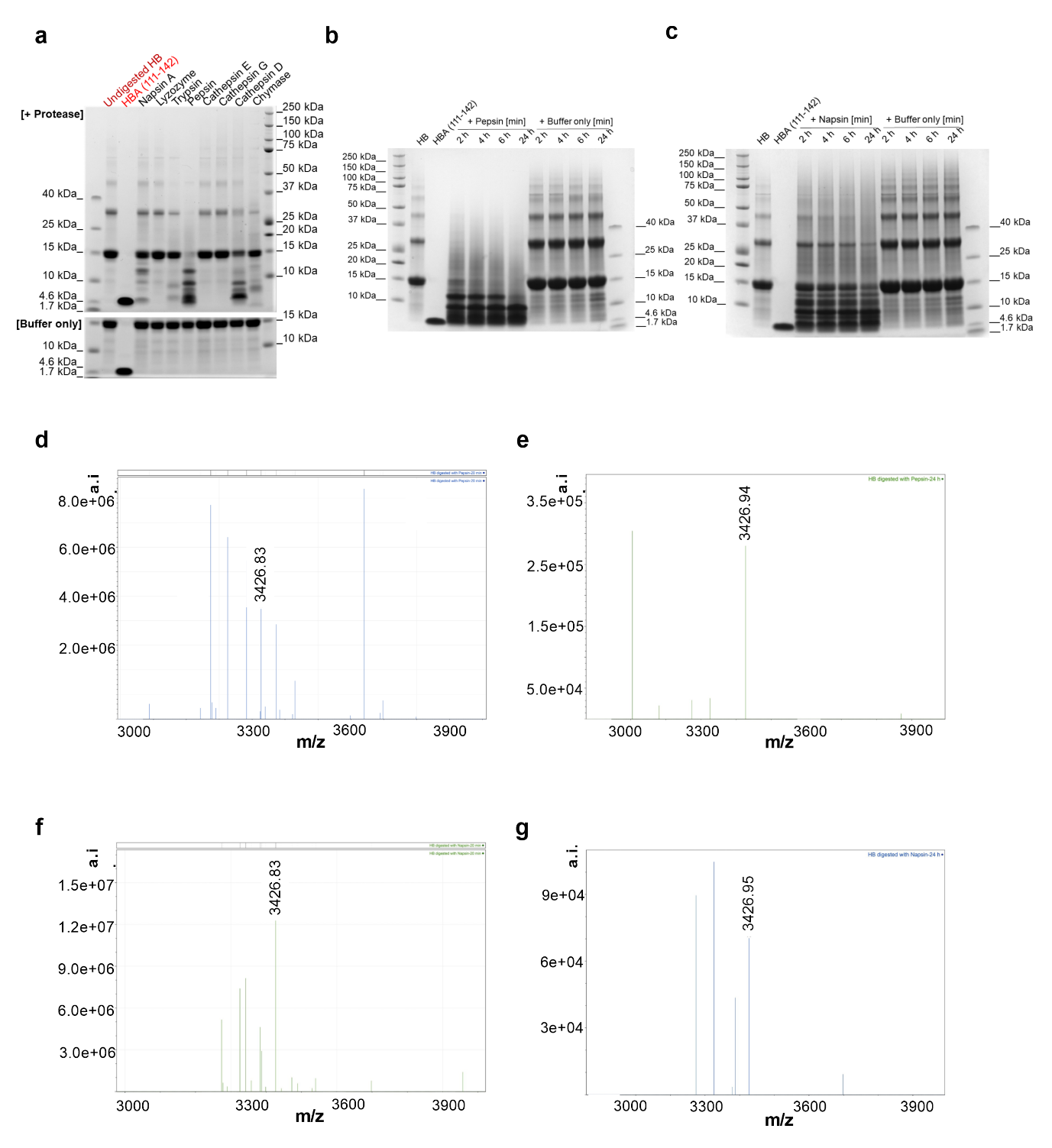
Fig. S3** **Generation of HBA(111-142) by proteolytic digestion.** **(a)** Purified human hemoglobin was incubated with several proteases (at 1:100 molar ratio) and digested for 2 h at 37 °C or incubated with the respective buffer only for the same time (bottom). Human hemoglobin was incubated with (b) pepsin (at pH 3.5) or (c) napsin A (at pH 3.6) at a 1:100 molar ratio, or with the respective digestion buffers without the addition of protease for 2, 4, 6, or 24 h at 37 °C. The reactions were separated by SDS-PAGE and total protein stained by colloidal Coomassie. To confirm the generation of HBA(111-142), full-length hemoglobin was incubated with **(d, e)** pepsin and **(f, g)** napsin A for 20 min (d, f) and 24 h (e, g) at 37 °C. Samples were then analyzed by LC-MS/MS, marked is the peak of generated HBA(111-142).

**
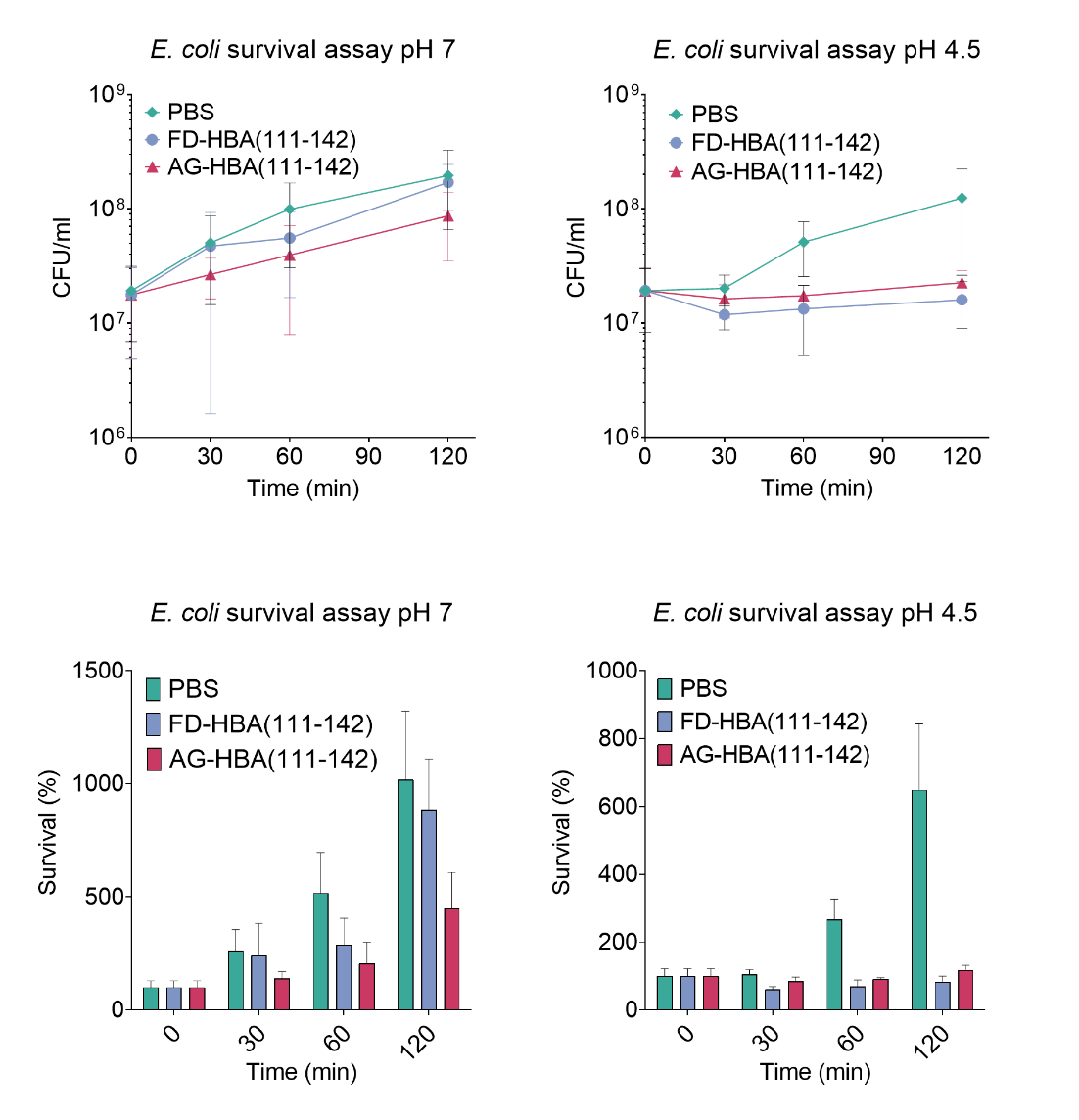
**

**Fig. S4 Effect of pH on the growth and survival of E. coli in the presence of HBA(111-142).** E. coli was incubated with 1 mg/ml FD- and AG-HBA(111-142) at pH 7 and 4.5. Bacterial survivability was quantified after 0, 30, 60, and 120 min by colony-forming units (CFU). The data in the two upper panels is not normalized, while the two lower panels show the same data, but each condition was normalized to the corresponding timepoint 0 that was set to 100%. Data were derived from three independent experiments and are shown as means ± SEM.


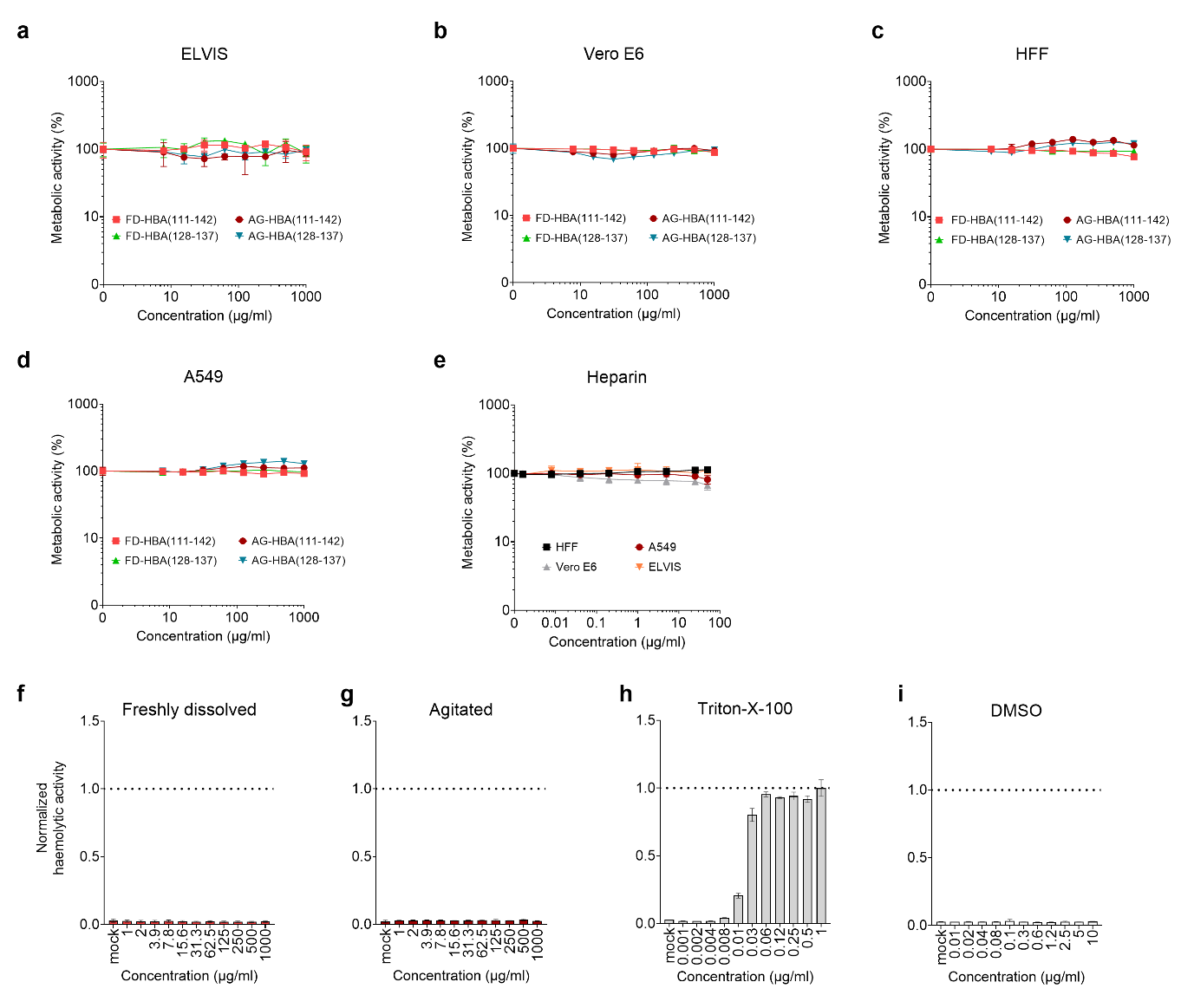


**Fig. S5** **Cytotoxic and hemolytic activity of HBA (111-142).** HBA(111-142) and HBA(128-137) either freshly dissolved (FD) or agitated (AG) **(a-d)** or heparin **(e)** were serially diluted in PBS, before the addition to the indicated cell lines. Two days post-addition, the metabolic activity of the cells was determined using the MTT assay. Values were baseline-subtracted and normalized to untreated cells. Shown is the mean $\pm$ SD of one experiment done in triplicates. HBA(111-142) either **(f)** FD or **(g)** AG were serially diluted in PBS and added to erythrocytes separated from human blood. After 1 h of co-incubation, the absorption of released hemoglobin in the supernatants was measured. **(h)** Triton-X was used as a positive control and **(i)** DMSO as a negative control. Full lysis of erythrocytes is represented by the 1% Triton-X-100 sample. Shown is the mean $\pm$ SD of one experiment done in triplicates.


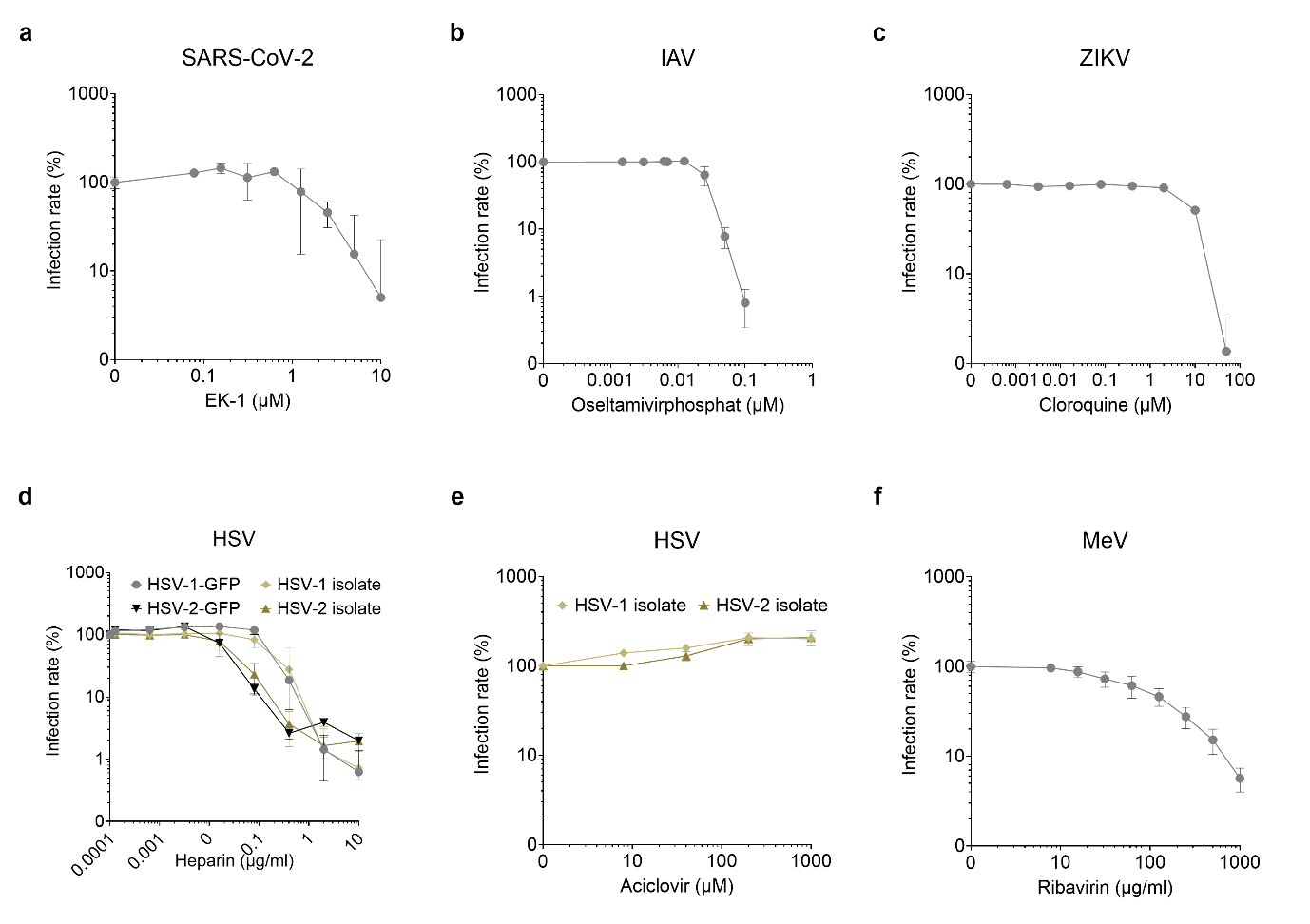


**Fig. S6** **Positive controls of virus infection inhibition**. Indicated virus inhibition controls were titrated in PBS, before the addition to the cells, resulting in the indicated final concentrations on cells. Following a 1 h incubation, target cells were infected with SARS-CoV-2, IAV, ZIKV, HSV-1, HSV-2, or MeV. Infection rates were determined one (HSV-1, HSV-2) or two days post-infection (all others). HSV-1 and HSV-2 isolates represent acyclovir-resistant strains. Values were baseline-subtracted and normalized to cells infected without the addition of compound. Shown are the means $\pm$ SD of one experiment performed in triplicates (SARS-CoV-2, IAV, ZIKV, MeV), or the mean of three independent experiments performed in triplicates $\pm$ SEM (HSV-1, HSV-2).

**
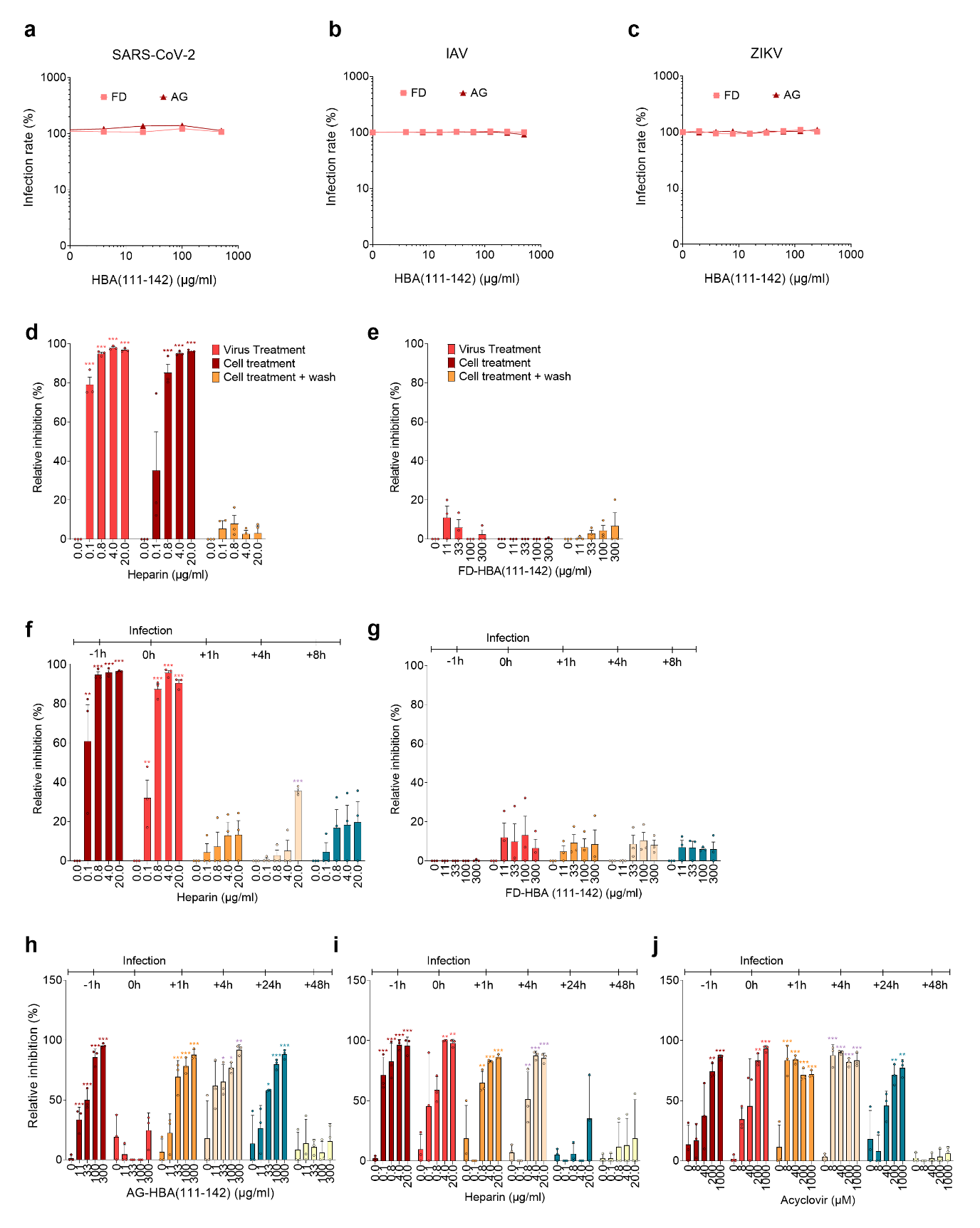
**

**Fig. S7** **Effect of HBA (111-142) on virus infection.** (**a-c)** HBA(111-142) either freshly dissolved (FD) or agitated (AG) was titrated in PBS, before adding it to the cells, resulting in the indicated final concentrations in cell culture. Following a 1 h incubation, respective target cells were infected with SARS-CoV-2, IAV, or ZIKV. Infection rates were determined two days post-infection. Experiments in d-g were performed in HFF cells. HSV-2 was exposed to freshly dissolved (FD) HBA(111-142) **(d)** or heparin **(e)** for 1 h, then these mixtures were used to infect cells (virus treatment). Alternatively, indicated concentrations of FD-HBA(111-142) or heparin were added to cells and incubated for 1 h before the cells were either directly infected with HSV-2 (cell treatment) or first washed, supplemented with fresh medium, and then infected (wash). FD-HBA(111-142) **(f)** or heparin **(g)** at indicated concentrations were added to the cells either 1 h prior to HSV-2 infection, at the time of infection (0 h) or 1, 4, or 8 h post-infection (with the removal of virus, washing, and addition of fresh medium). Infection rates were determined one day post-infection by quantifying the virus expression of virus-encoded –GFP using flow cytometry. Experiments in h-j were performed in ELVIS cells: AG-HBA(111-142) **(h)** heparin **(i)**, or acyclovir **(j)** at indicated concentrations were added to the cells either 1 h prior to HSV-2 infection, at the time of infection (0 h) or 1, 4, 24, or 48 h post-infection (with the removal of virus, washing, and addition of fresh medium). Infection rates were determined 49 h post-infection by measuring enzyme activity of the ELVIS reporter cell line. In all cases, concentrations represent final concentration of the compound in the cell culture. Values were baseline-subtracted and normalized to cells infected without the addition of compound (a-c). Values shown in d-j representing relative inhibition in % were normalized to cells infected without the addition of the compound. Shown are the means of three independent experiments ± SEM (d-g), or the means of three independent experiments ± SD (a-c; h-j) each performed in triplicates. Significant differences were determined by one-way ANOVA followed by Bonferroni’s multiple comparison test. *p< 0.033, **p< 0.002, ***p<0.0002.

**
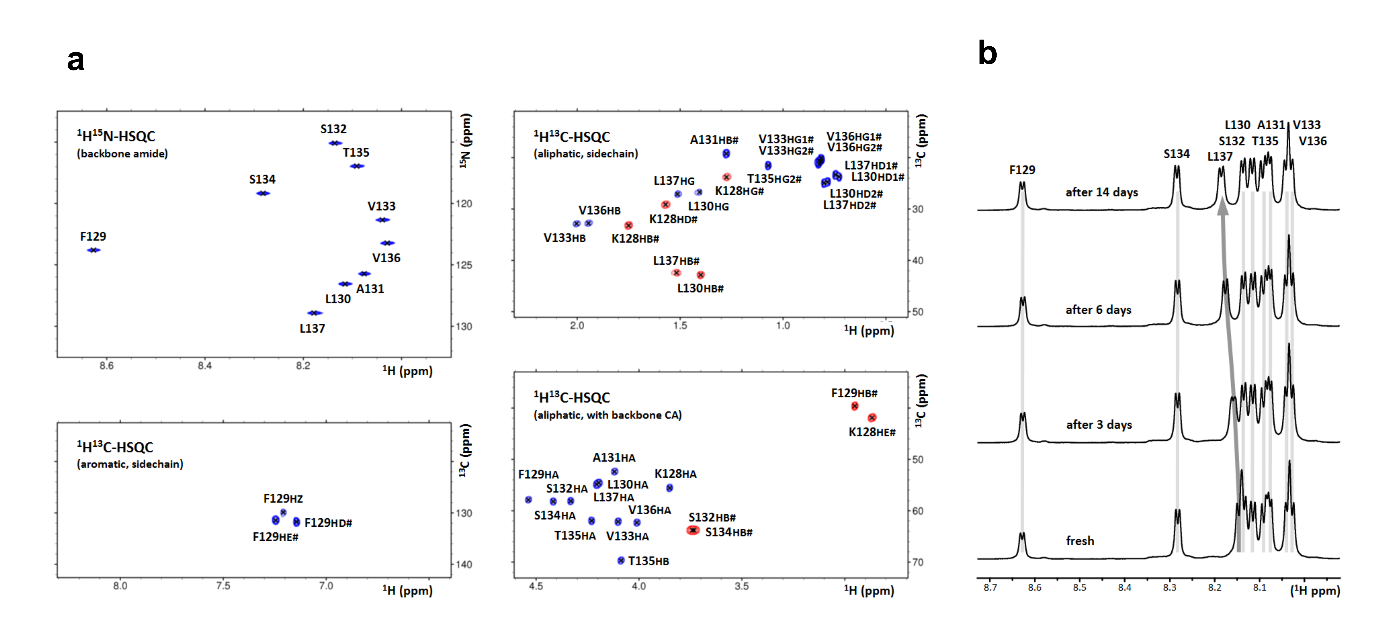
Fig. S8 NMR spectra of the HBA(128-137) 10-mer peptide. (a)** 2D ^1^H^15^N-HSQC and 2D ^1^H^13^C-HSQC spectrum: the amide (HN) backbone resonance assignment is indicated in the 2D ^1^H^15^N-HSQC (top left) and three sections of the 2D ^1^H^13^C-HSQC spectrum show the aliphatic and aromatic regions in which the backbone CA and subsequent sidechain resonance assignments are indicated. Due to multiplicity editing, the CH_2_ groups have a different sign (red) than the CH and CH_3_ groups (blue). **(b)** ^1^D ^1^H NMR spectra: 10-mer peptide measured after several days showing perturbation of the C-terminal Leucine (L137) backbone amide signal.

**Supplementary Tables:**

**Table S1. Residue-wise SASA using the complete trajectories (300 ns).** Average over the three replicas, with standard deviations between parenthesis in Å^2^ for the proposed recognition site (108-113) at pH 7.4 and pH 3.6

|  |  | | | | | |
| --- | --- | --- | --- | --- | --- | --- |
| **Residue No.** | **108** | **109** | **110** | **111** | **112** | **113** |
| **pH 7.4** | 0.23  (0.42) | 5.58  (3.15) | 0.15  (0.28) | 8.44  (5.58) | 72.06  (17.79) | 31.33  (10.98) |
| **pH 3.6** | 48.85  (38.13) | 42.69  (35.27) | 24.49  (27.06) | 36.65  (23.92) | 91.70  (53.90) | 49.85  (36.46) |

**Table S2. Properties and antiviral activity of HBA(111-142) length fragments**

| **HBA** | **Sequence** | **Mass**  **(g/mol)** | **pI^1^** | **a3vSA^2^** | **ThT^3^ signal (+/-)** | **IC_50_^4^**  **(µg/ml ±SD)** |
| --- | --- | --- | --- | --- | --- | --- |
| 111-142 | AAHLPAEFTPAVHASL**DK**FLASVSTVLTS**K**Y**R** | 3429 | 9.72 | 0.08 | + | 89 (± 17.7) |
| 111-137 | AAHLPAEFTPAVHASL**DK**FLASVSTVL | 2793 | 6.05 | 0.13 | + | 130 (± 14.4) |
| 111-131 | AAHLPAEFTPAVHASL**DK**FLA | 2206 | 6.05 | 0.2 | - | > 130 |
| 118-142 | FTPAVHASL**DK**FLASVSTVLTS**K**Y**R** | 2739 | 10.2 | 0.22 | + | 71.5 (± 12) |
| 132-142 | SVSTVLTS**K**Y**R** | 1240 | 10.41 | 0.21 | - | > 130 |
| 127-137 | **DK**FLASVSTVL | 1179 | 6.66 | 0.38 | + | 19.5 (± 12.1) |
| 132-137 | SVSTVL | 605 | 3.37 | 0.64 | - | > 130 |
| 128-137 | **K**FLASVSTVL | 1067 | 9.91 | 0.59 | + | 19.3 (± 3.5) |
| 128-136 | **K**FLASVSTV | 951 | 5.52 | 0.76 | - | > 130 |
| 128-135 | **K**FLASVST | 851 | 9.91 | 0.37 | - | > 130 |
| 129-137 | FLASVSTVL | 936 | 5.52 | 0.76 | - | > 130 |
| 130-137 | LASVSTVL | 788 | 5.52 | 0.64 | + | > 130 |
| 131-137 | ASVSTVL | 675 | 5.57 | 0.54 | + | > 130 |

1: pI, isoelectric point; 2: a3vSA, average of the aggregation propensity values as calculated by the AGGRESCAN software[29]; 3: ThT, formation of ThT positive aggregates (+/-); 4: Antiviral activity; Serial dilutions of peptides were added to ELVIS cells, which were infected 1 h later with HSV-2; infection rates were determined one day post-infection by measuring lacZ enzyme activity. IC_50_ values were derived from one (inactive peptides) to three (antivirally active peptides) experiments performed in triplicates.

**Table S3. Resonance assignment (^1^H, ^13^C and ^15^N in ppm) of the HBA(128-137) 10-mer peptide.** The used NMR spectra are referenced based on the signal of water (4.7 ppm)


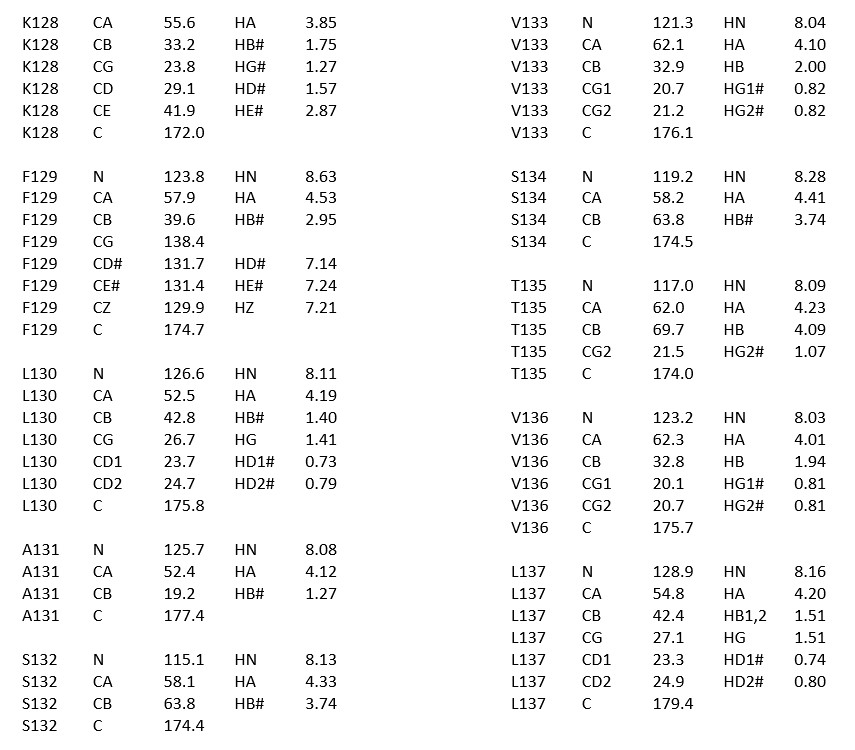


**Table S4. Structure statistics for the HBA(128-137) 10-mer peptide.** The number of experimental restraints (*) for the structure calculation is given as provided per monomer unit. The Ramachandran score (**) and the average rmsd to mean values (***) are calculated either for the whole ensemble of 3 × 20 structures and for the middle monomer strand (by using Procheck and Molmol respectively)


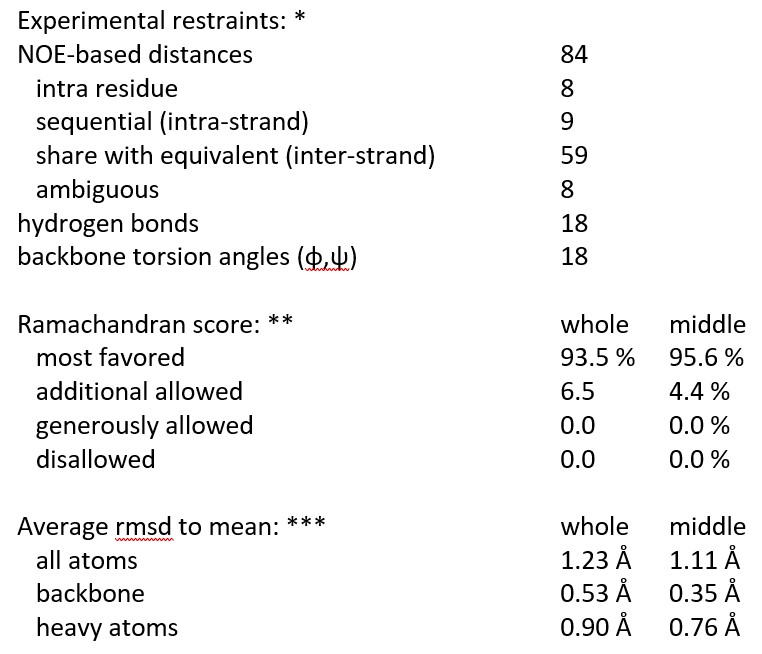


**Additional References**

1. Park S-Y, Yokoyama T, Shibayama N, et al (2006) 1.25 Å Resolution Crystal Structures of Human Haemoglobin in the Oxy, Deoxy and Carbonmonoxy Forms. J Mol Biol 360:690–701. https://doi.org/10.1016/j.jmb.2006.05.036

2. Anandakrishnan R, Aguilar B, Onufriev A v. (2012) H++ 3.0: automating pK prediction and the preparation of biomolecular structures for atomistic molecular modeling and simulations. Nucleic Acids Res 40:W537–W541. https://doi.org/10.1093/nar/gks375

3. Case DA, Belfon K, Ben-Shalom IY, Brozell SR, Cerutti DS, Cheatham TE, Cruzeiro VWD, Darden TA, Duke RE, Giambasu G, Gilson MK, Gohlke H, Goetz AW, Harris R, Izadi S, Izmailov SA, Kasavajhala K, Kovalenko A, Krasny R, Kurtzman T, Lee TS, LeGrand S, Li P, Lin C, Liu J, Luchko T, Luo R, Man V, Merz KM, Miao Y, Mikhailovskii O, Monrad G, Nguyen H, Onufriev A, Pan F, Pantano S, Qi R, Roe DR, Roitberg A, Sagui C, Schott-Verdugo S, Shen J, Simmerling CL, Skrynnikov NR, Smith J, Swails J, Walker RC, Wang J, Wilson L, Wolf RM, Wu X, Xiong Y, Xue Y, York DM, and Kollman PA (2020), AMBER 2020, University of California, San

4. Giammona DA (1984) Parameters modified by Case and Bayly. University of California

5. Maier JA, Martinez C, Kasavajhala K, et al (2015) ff14SB: Improving the Accuracy of Protein Side Chain and Backbone Parameters from ff99SB. J Chem Theory Comput 11:3696–3713. https://doi.org/10.1021/acs.jctc.5b00255

6. Jorgensen WL, Chandrasekhar J, Madura JD, et al (1983) Comparison of simple potential functions for simulating liquid water. J Chem Phys 79:926–935. https://doi.org/10.1063/1.445869

7. Darden T; York D; Pedersen L Particle mesh Ewald: An N·log(N) method for Ewald sums in large systems. J. Chem. Phys 1993, 98, 10089–10092.

8. Miao Y, Feher VA, McCammon JA (2015) Gaussian Accelerated Molecular Dynamics: Unconstrained Enhanced Sampling and Free Energy Calculation. J Chem Theory Comput 11:3584–3595. https://doi.org/10.1021/acs.jctc.5b00436

9. Roe DR, Cheatham TE (2013) PTRAJ and CPPTRAJ: Software for Processing and Analysis of Molecular Dynamics Trajectory Data. J Chem Theory Comput 9:3084–3095. https://doi.org/10.1021/ct400341p

10. Shao J, Tanner SW, Thompson N, Cheatham TE (2007) Clustering Molecular Dynamics Trajectories: 1. Characterizing the Performance of Different Clustering Algorithms. J Chem Theory Comput 3:2312–2334. https://doi.org/10.1021/ct700119m

11. Hubbard S, Thornton J (1993) NACCESS, Computer Program

12. Noschka R, Wondany F, Kizilsavas G, et al (2021) Gran1: A Granulysin-Derived Peptide with Potent Activity against Intracellular Mycobacterium tuberculosis. Int J Mol Sci 22:8392. https://doi.org/10.3390/ijms22168392

13. Folch J, Lees M, Sloane GH A SIMPLE METHOD FOR THE ISOLATION AND PURIFICATION OF TOTAL LIPIDES FROM ANIMAL TISSUES*

14. Weil T, Groß R, Röcker A, et al (2020) Supramolecular Mechanism of Viral Envelope Disruption by Molecular Tweezers. J Am Chem Soc 142:17024–17038. https://doi.org/10.1021/jacs.0c06400

15. Proffitt MR, Schindler SA (1995) Rapid detection of HSV with an enzyme-linked virus inducible system^TM^ (ELVIS^TM^) employing a genetically modified cell line. Clin Diagn Virol 4:175–182. https://doi.org/10.1016/0928-0197(95)00011-V

16. REED LJ, MUENCH H (1938) A SIMPLE METHOD OF ESTIMATING FIFTY PER CENT ENDPOINTS12. Am J Epidemiol 27:493–497. https://doi.org/10.1093/oxfordjournals.aje.a118408

17. Andreoni M, Faircloth M, Vugler L, Britt WJ (1989) A rapid microneutralization assay for the measurement of neutralizing antibody reactive with human cytomegalovirus. J Virol Methods 23:157–167. https://doi.org/10.1016/0166-0934(89)90129-8

18. Braun E, Hotter D, Koepke L, et al (2019) Guanylate-Binding Proteins 2 and 5 Exert Broad Antiviral Activity by Inhibiting Furin-Mediated Processing of Viral Envelope Proteins. Cell Rep 27:2092-2104.e10. https://doi.org/10.1016/j.celrep.2019.04.063

19. Conzelmann C, Gilg A, Groß R, et al (2020) An enzyme-based immunodetection assay to quantify SARS-CoV-2 infection. Antiviral Res 181:104882. https://doi.org/10.1016/j.antiviral.2020.104882

20. Dick GWA, Kitchen SF, Haddow AJ (1952) Zika Virus (I). Isolations and serological specificity. Trans R Soc Trop Med Hyg 46:509–520. https://doi.org/10.1016/0035-9203(52)90042-4

21. Mor G, Kwon J-Y (2015) Trophoblast-microbiome interaction: a new paradigm on immune regulation. Am J Obstet Gynecol 213:S131–S137. https://doi.org/10.1016/j.ajog.2015.06.039

22. Karsi A, Lawrence ML (2007) Broad host range fluorescence and bioluminescence expression vectors for Gram-negative bacteria. Plasmid 57:286–295. https://doi.org/10.1016/j.plasmid.2006.11.002

23. Chandra A, Singh N (2018) Bacterial growth sensing in microgels using pH-dependent fluorescence emission. Chemical Communications 54:1643–1646. https://doi.org/10.1039/C7CC08678D

24. Brünger AT, Adams PD, Clore GM, et al (1998) Crystallography &amp; NMR System: A New Software Suite for Macromolecular Structure Determination. Acta Crystallogr D Biol Crystallogr 54:905–921. https://doi.org/10.1107/S0907444998003254

25. Linge JP, O’Donoghue SI, Nilges M (2001) Automated Assignment of Ambiguous Nuclear Overhauser Effects with ARIA. pp 71–90

26. Goddard D, Kneller DG (2000) SPARKY3

27. Shen Y, Bax A (2013) Protein backbone and sidechain torsion angles predicted from NMR chemical shifts using artificial neural networks. J Biomol NMR 56:227–241. https://doi.org/10.1007/s10858-013-9741-y

28. Linge JP, Williams MA, Spronk CAEM, et al (2003) Refinement of protein structures in explicit solvent. Proteins: Structure, Function, and Bioinformatics 50:496–506. https://doi.org/10.1002/prot.10299

29. Conchillo-Solé O, de Groot NS, Avilés FX, et al (2007) AGGRESCAN: a server for the prediction and evaluation of “hot spots” of aggregation in polypeptides. BMC Bioinformatics 8:65. https://doi.org/10.1186/1471-2105-8-65

30. Zandomeneghi G, Krebs MRH, McCammon MG, Fändrich M (2009) FTIR reveals structural differences between native β-sheet proteins and amyloid fibrils. Protein Science 13:3314–3321. https://doi.org/10.1110/ps.041024904

31. Zhang J, Xin L, Shan B, et al (2012) PEAKS DB: De Novo Sequencing Assisted Database Search for Sensitive and Accurate Peptide Identification. Molecular & Cellular Proteomics 11:M111.010587. https://doi.org/10.1074/mcp.M111.010587
